# Novel EG95-based mRNA vaccines induce long-term protective immunity against echinococcosis

**DOI:** 10.1371/journal.ppat.1014659

**Published:** 2026-10-09

**Authors:** Wei-Gang Chen, Li Li, Guo-Dong Dai, Yao-Dong Wu, Chuan-Gang Zhu, Huan Zhang, Xiu-Rong Li, Le Li, Nian-Zhang Zhang, Li-Qun Wang, Guo-Cai Li, Wen-Ling Ma, Ji-Tao Pan, Ming-Lu Tan, Fu-Lan Da, Hong Yin, Bao-Quan Fu, Xue-Nong Luo, Hong-Bin Yan, Wan-Zhong Jia

**Affiliations:** 1 State Key Laboratory of Animal Disease Control and Prevention, College of Veterinary Medicine, Lanzhou University, National Professional Laboratory for Animal Echinococcosis, Gansu Province Research Center for Basic Disciplines of Pathogen Biology, Key Laboratory of Veterinary Parasitology of Gansu Province, Key Laboratory of Veterinary Etiological Biology and Key Laboratory of Ruminant Disease Prevention and Control, Ministry of Agricultural and Rural Affairs, Lanzhou Veterinary Research Institute, Chinese Academy of Agricultural Sciences, Lanzhou, Gansu Province, China; 2 Key Laboratory of Animal Parasitology, Ministry of Agriculture/ Shanghai Veterinary Research Institute, Chinese Academy of Agricultural Sciences, Shanghai, China; 3 Huangyuan County Animal Husbandry and Veterinary Station, Xining, Qinghai Province, China; 4 Animal Science Department, Qinghai Agricultural and Animal Husbandry Technology Vocational College, Xining, Qinghai Province, China; 5 Jiangsu Co-innovation Center for Prevention and Control of Important Animal Infectious Disease, Yangzhou, Jiangsu Province, China; CSIRO ACDP: CSIRO Australian Centre for Disease Preparedness Business Unit, AUSTRALIA

## Abstract

Cystic echinococcosis (CE), a neglected zoonotic disease caused by *Echinococcus granulosus* sensu lato*,* remains a significant public health and veterinary concern worldwide. Despite efforts to develop recombinant protein-based vaccines against EG95, a key protective antigen, existing platforms suffer from limited immunogenicity, short-lived immunity, and complex production processes. Here, we designed and evaluated three novel mRNA-LNP vaccines, including EG95 mRNA-LNP vaccine, IL-4-EG95 mRNA-LNP vaccine encoding EG95 fused with IL-4 via a flexible linker to enhance immune stimulation, and EG95-EC95 mRNA-LNP vaccine that multivalent formulation incorporating EC95 antigens from *Echinococcus canadensis*. The vaccine candidates were efficiently encapsulated in lipid nanoparticles and demonstrated robust target expression in vitro and in vivo. Immunization induced significantly stronger, more durable humoral and cellular immune responses compared to a commercial EG95 subunit vaccine both in mice and lambs. Notably, high levels of specific antibodies persisted for over 12 months post-vaccination. Parasites infection challenge experiments confirmed that these three mRNA-LNP vaccines, especially the IL-4-EG95 vaccine conferred superior protection, inhibited protoscolex survival and cyst growth. These results highlight the advantages of mRNA-based vaccines in safety, scalability, and flexibility over conventional approaches, offering a promising strategy for controlling *E. granulosus* sensu lato transmission and reducing the zoonotic burden of CE.

## Author summary

*Echinococcus granulosus* causes a severe zoonotic disease with substantial clinical and economic burdens across endemic regions. Current vaccine strategies relying on protein subunits remain limited by poor immunogenicity and short-lived protection. Here, we establish a novel mRNA-LNP platform delivering optimized EG95-based antigens that elicit balanced Th1/Th2 immunity and high-level, durable antibody responses. Our IL-4-EG95 candidate conferred advantageous protection in mice, significantly outperforming a commercial subunit vaccine. This study demonstrates the potential of mRNA-based vaccines to overcome key limitations of conventional anti-parasitic vaccines and opens a versatile path for controlling genetically diverse parasitic pathogens.

## Introduction

Cystic echinococcosis (CE) is a serious zoonotic parasitic infection caused by the metacestode stage of *Echinococcus granulosus* sensu lato [1,2]. The disease is highly endemic in pastoral communities, where it substantially impacts livestock productivity and poses a major public health risk. In recognition of its neglect and significant burden, the World Health Organization has included CE among the 17 neglected tropical diseases [3,4]. Control of CE primarily relies on interrupting the transmission cycle of *E. granulosus* sensu lato between canine (definitive hosts) and livestock (intermediate hosts) [5]. It is widely established that the most effective control strategy involves preventing the development of parasite eggs into viable oncospheres [6], thereby blocking subsequent establishment, proliferation, and cyst formation in intermediate hosts. This intervention ultimately reduces transmission of the parasite from dogs to humans [7]. Immunization with effective vaccines represents a pivotal strategy for achieving this objective [8–11].

EG95 is a well-characterized vaccine antigen originally identified from *Echinococcus granulosus* oncospheres. It is encoded by a member of the EG95 gene family [12], and the gene product has been localized specifically to the penetration glands of the infective larval (oncosphere) stage [13]. These penetration glands secrete proteases and other molecules that facilitate the parasite's invasion through the intestinal wall and migration to target organs [13]. The EG95 protein is also conserved in *Echinococcus multilocularis* (where the homologue is termed EM95), sharing approximately 80% amino acid sequence identity with its *E. granulosus* counterpart [14,15]. Vaccination with EG95/EM95 induces antibodies that target these secreted components, thereby neutralizing the oncosphere's ability to establish infection in the intermediate host [14]. This mechanism blocking the early establishment phase before cyst development explains why targeting EG95 can achieve high protective efficacy (96–100% in sheep for *E. granulosus*; 78–83% in mice for *E. multilocularis*) [14,16].

Recent studies have systematically explored recombinant EG95 protective antigen expressed in various systems, including viral vectors such as *Orf* virus [17], *rabies* virus [18] and *small ruminant morbillivirus* [19], bacterial hosts such as *Escherichia coli* (BL21) [20] and *Lactococcus lactis* [21], as well as other platforms including ferritin-based nanoparticles [22] and minigene vaccines [23]. However, the EG95 commercial subunit vaccine candidates exhibit notable limitations, such as limited duration of protection, and suboptimal response to mutant strains, cumbersome production processes, and inadequate purification leading to impurity concerns [24]. In contrast, a recent study developed an EG95-based DNA vaccine [24], which incorporating IL-2, and through concurrent optimization of lipid nanoparticle delivery and vaccination schedules, demonstrated that the formulation (RNP/pEG95-IL-2) induces superior and sustained humoral and cellular immunity in mice compared to a commercial vaccine, underscoring the advantages of nucleic acid platforms for immunogenicity and manufacturing. This novel approach shows significantly improved immunogenicity and a more efficient manufacturing process compared to conventional subunit vaccines.

The concept of mRNA-based vaccines was first proposed over three decades ago, motivated by the promise of a safe, versatile, and easily producible vaccine platform [25,26]. During the SARS-CoV-2 pandemic, mRNA-based vaccines proved highly effective, accelerating the development and deployment of mRNA technology at an unprecedented pace [27,28]. This progress has spurred comprehensive advances in mRNA vaccine research against major pathogens such as influenza virus [29], Zika virus [30], rabies virus [31], and Plasmodium [32], *Toxoplasma gondii* [33,34], *Leishmania donovani* [35], resulting in significantly enhanced immunogenicity. Moreover, the streamlined production process particularly in the design and assembly stages has greatly reduced the complexity and effort previously associated with vaccine development. Principally, mRNA vaccines offer several advantages over conventional vaccine platforms. Unlike some viral vector or DNA-based vaccines, mRNA does not carry the risk of integration into the host genome, thereby alleviating concerns regarding insertional mutagenesis [36]. Furthermore, mRNA vaccines can be manufactured in a cell-free manner, enabling rapid, scalable, and cost-effective production. Additionally, a single mRNA vaccine can encode multiple antigens, enhancing the immune response against complex pathogens and enabling multiplex targeting [27,36,37].

Therefore, building on the advancements in both echinococcosis vaccine research and mRNA technology, we aimed to develop an mRNA-based vaccine targeting *E. granulosus*. Here, we designed a mRNA-based vaccine expressing a fusion protein comprising the EG95 protective antigen and IL-4 immunostimulatory factor connected via a flexible linker to ensure proper protein folding and preserve immunological activity. IInterleukin-4 (IL-4), a hallmark cytokine of type 2 inflammation, serves as a key mediator in host responses to parasitic infections and allergens. It promotes Th2 lineage commitment in naïve CD4 ⁺ T cells while simultaneously driving immunoglobulin class switching to IgG1 and IgE in B cells, thereby orchestrating the development of Th2-type immunity [38]. Besides, given the global distribution and genetic diversity of *E. granulosus* [39], we simultaneously adopted a multivalent vaccine strategy combined EG95 and EC95 immunogenic antigens from multiple genotypes. EC95 is a potential antigen against *E. canadensis* (genotype G6) — the second prevalent genotype in the global [40] was selected as a target for the combined immunization approach. Lipid nanoparticles (LNPs) have emerged as a transformative platform to circumvent the limitations of conventional drug formulations, offering reduced systemic toxicity, enhanced targeted delivery, improved drug permeability, and controlled sustained release. In addition, LNPs hold considerable promise for unlocking the antiparasitic potential of natural products [41,42].The success of mRNA vaccines is fundamentally based on LNPs, which enable efficient mRNA expression coupled with intrinsic adjuvant properties that drive potent antibody responses. Advances in LNP technology have made it possible to overcome challenges related to mRNA stability, immunogenicity, and targeted delivery. Optimization of LNP composition enhances mRNA uptake by diverse splenic immune cell populations, thereby promoting robust antigen presentation and overall vaccine efficacy [43–45], so the mRNA was encapsulated in lipid nanoparticles (LNPs) for efficient delivery. Then we assessed target gene expression both in vitro and in vivo, as well as the resulting humoral and cellular immune responses. Furthermore, a challenge experiment was conducted to evaluate the protective efficacy of the mRNA vaccine against infection.

## Results

### Design and assembly of mRNA vaccines

The EG95 protein, known for its high conservation and strong immunogenicity, represents a promising vaccine target against cystic echinococcosis. Based on the EG95 framework, we designed three structurally optimized mRNA constructs (Fig 1A), For IL-4-EG95 mRNA, IL-4 was added and incorporated at the N-terminus, to enhance antigen-stimulated B cell immunogenicity and promote Th2 cell differentiation. For EG95-EC95 mRNA, EC95 was genetically linked to EG95 to emulate a combined immunogenic structure, thereby enabling the coordinated presentation of multiple antigens from a single vector to synergistically stimulate the immune system, with a flexible linker inserted between the two proteins to ensure proper folding and expression. For EG95 mRNA, the construct encodes the native EG95 antigen.

**Fig 1:**
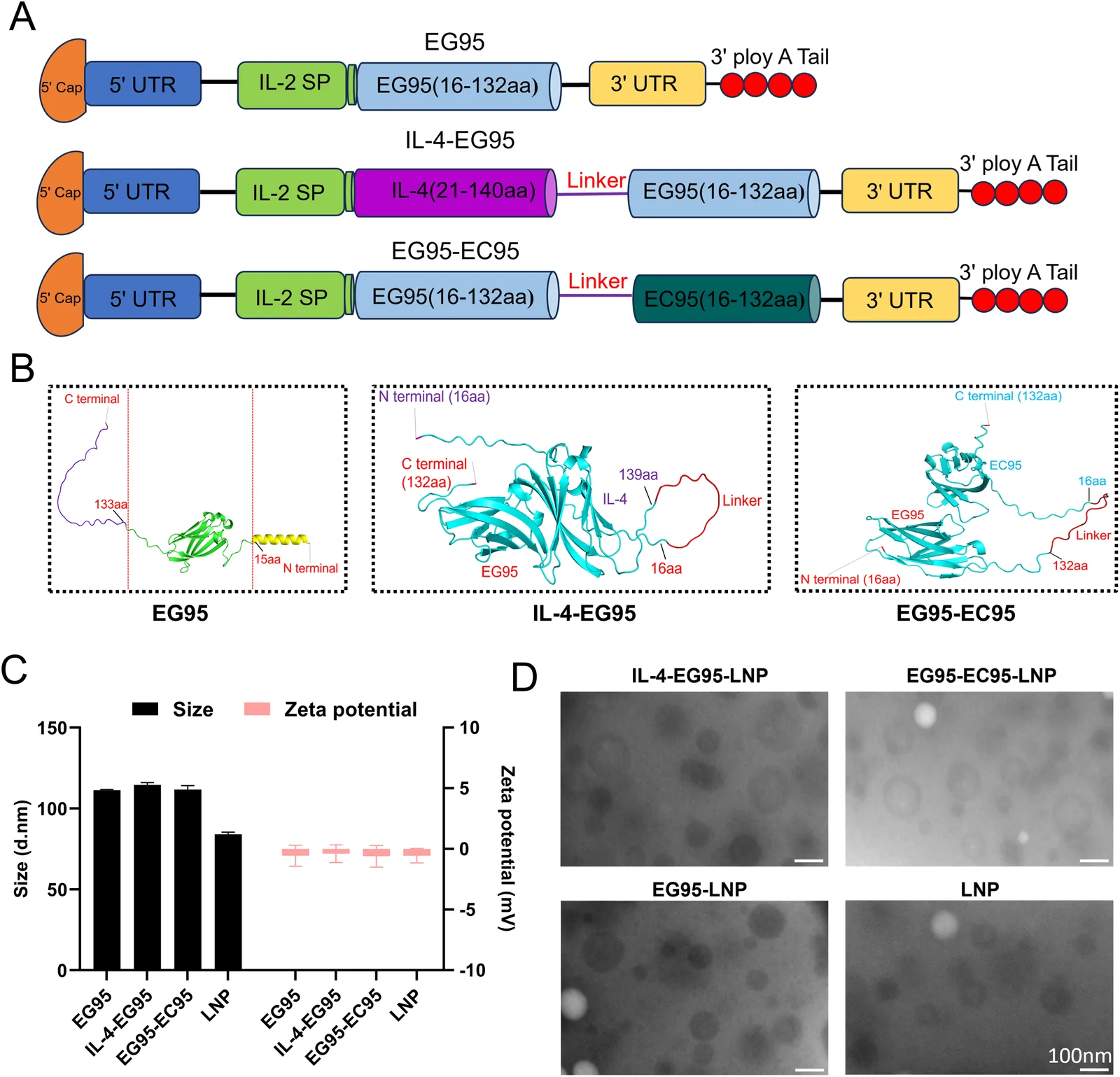
Construction of the expression vector and physicochemical characterization of the three LNP-based mRNA vaccines. **(A)** Schematic representation of the mRNA construct of the vaccines, comprising the engineered potent antigen EG95, EC95, the immunostimulatory adjuvant IL-4, a secretory signal peptide, and a flexible linker. **(B)** Prediction of the tertiary structure of the expressed recombinant proteins of the vaccines, the antigen (EG95/EC95) was engineered to remove its native signal peptide and transmembrane region. **(C)** Physicochemical characterization of LNP-mRNA lipoplexes. Size and zeta potential of various lipoplexes LNP-mRNA binary complexes. **(D)** Structural analysis of LNP formulations via TEM. Scale bars: 100 nm. Data are presented as means ± standard deviation (SD, n = 3).

To enable high-level expression, each mRNA construct was engineered to include a 5′ Cap 1 structure (N7mGpppAm), optimized 5′ and 3′ untranslated regions (UTRs), a codon-optimized open reading frame (ORF) encoding the target antigen. And a poly(A) tail was incorporated to enhance mRNA stability and translational efficiency. The predicted tertiary structures of the resulting recombinant proteins were shown in Fig 1B. After confirming plasmid sequences by DNA sequencing, large-scale plasmid preparations were used for in vitro transcription (IVT) with T7 RNA polymerase. The resulting mRNA was encapsulated into LNP with molar ratios of 50:10:1.5:38.5 (ionizable lipid T19: DSPC: DMG-PEG2000: cholesterol). The mRNA-LNP formulations exhibited uniform size distribution, with diameters ranging from 110 to 116 nm and a low polydispersity index (PDI) between 0.08 and 0.1 (Fig 1C), indicated optimal physical characteristics for cellular uptake and biodistribution. The empty LNPs exhibited a uniform distribution with particle sizes ranging from 82 nm to 85 nm. The encapsulation efficiency of the resulting mRNA-LNPs was determined to be above 95% (S2 Table). Both empty and mRNA-loaded LNPs displayed spherical shapes and homogeneous distribution as observed by TEM (Fig 1D).

### Gene delivery efficiency of mRNA vaccines in vitro

The efficacy of mRNA vaccines depends critically on the level of antigen expression in vivo. To elucidate this mechanism, we transfected cells with mRNA constructs using a commercial transfection reagent. Transfection efficiency was quantified via quantitative reverse transcription PCR (qRT-PCR) to measure cellular EG95 mRNA levels (Fig 2A). All three mRNA constructs exhibited high expression levels in cells, with a clear time-dependent increase were observed. Notably, the IL-4-EG95 fusion demonstrated significantly higher expression than the other two molecular variants (*P* < 0.01). The specificity and size of the quantitative real-time PCR products were further confirmed by agarose gel electrophoresis (Fig 2B).

**Fig 2:**
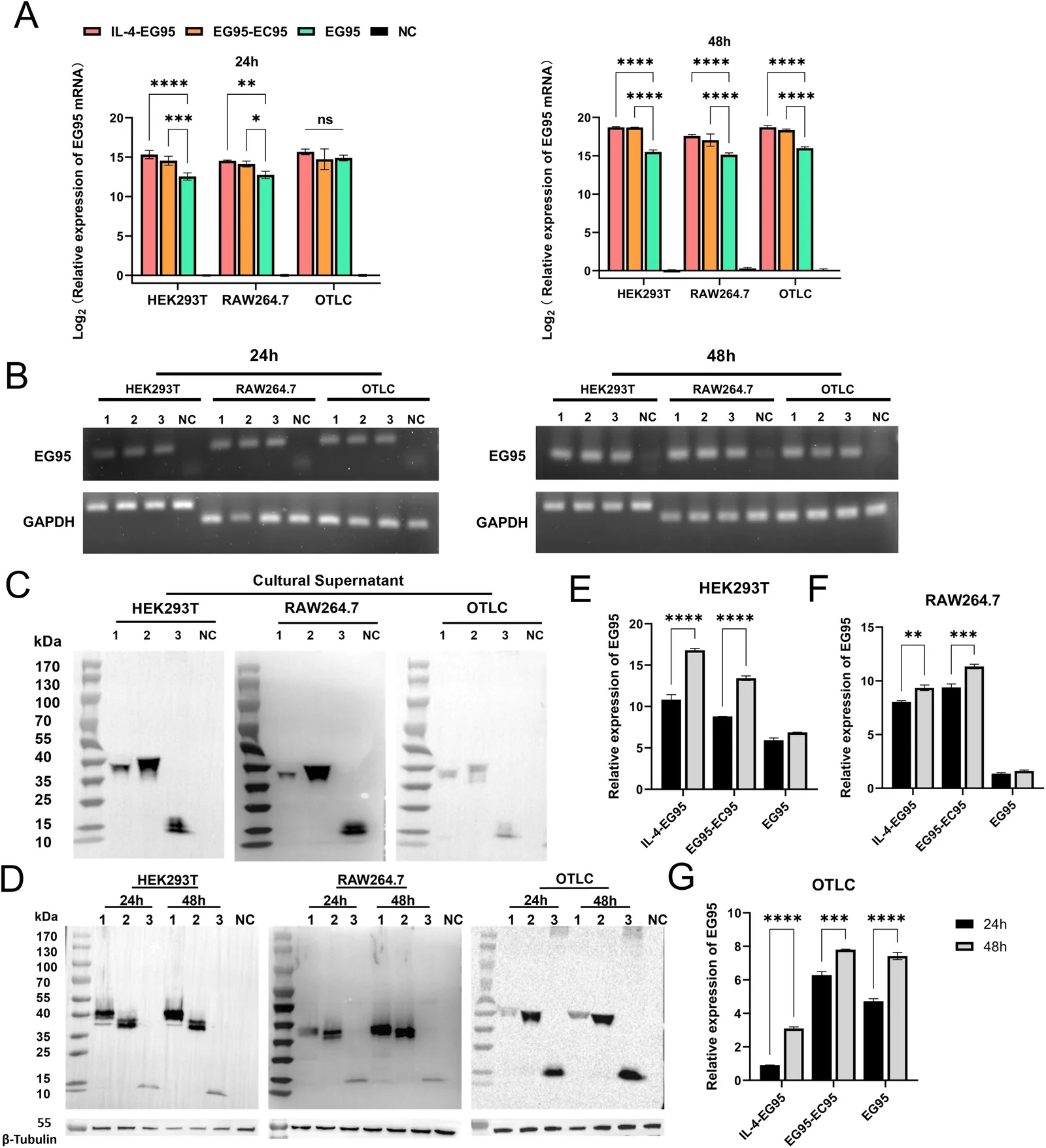
Gene delivery efficiency of mRNA vaccine in vitro. **(A)** HEK293T, RAW264.7, and OTLC cells were transfected with mRNA-transfection reagent complexes and harvested at 24 h and 48 h post-transfection. EG95 mRNA levels in HEK293T, RAW264.7, and OTLC cells were quantified by qPCR normalized to GAPDH. **(B)** Agarose gel electrophoresis of EG95 qPCR products in HEK293T, RAW264.7, and OTLC cells. GAPDH served as the reference gene (Lane1 ~ 3: IL-4-EG95, EG95-EC95, EG95; Lane 4: NC, negative control). Western blot analysis of EG95 protein in cell culture supernatants (**C**) and cells (**D**) at 24 and 48 hours, respectively. Western blot showing the relative expression levels of EG95 protein in HEK293T **(E)**, RAW264.7 **(F)**, and OTLC (**G**) cell lysates. Protein quantification was performed with normalization to β-tubulin as the loading control. Data are presented as means ± SD (n = 3), * *P* < 0.05, ** *P* < 0.01, *** *P* < 0.001, **** *P* < 0.0001.

Cells and cell culture supernatants were harvested at 24, 48 hours post-transfection for Western blot analysis. The results confirmed successful transcription of all three mRNA constructs across three different cell types (Fig 2C), leading to synthesis of the target proteins-IL-4-EG95 (38 kDa), EG95-EC95 (36 kDa) and EG95(15 kDa). Protein expression increased in a time-dependent manner. Detection of the target protein in the supernatants indicated that all mRNA constructs supported full gene expression from transcription and translation to secretion in cellular environments (Fig 2D). The similar results were observed in HEK293T and RAW264.7 cells, the protein expression levels in IL-4-EG95 and EG95-EC95 mRNA transfected cells were elevated compared to EG95 treated cells (Fig 2E,2F), whereas OTLC cells demonstrated high-level expression of the EG95 construct (Fig 2G).

After incubated with three mRNA-LNPs, cytoplasmic fluorescence signals were observed in all cell types, confirmed that LNP-encapsulated nanoparticles successfully delivered mRNA into cells and facilitated functional protein production. The delivery efficiency of mRNA-LNP was notably higher in RAW264.7 (Fig 3B) and OTLC cells (Fig 3C), while HEK293T cells (Fig 3A) exhibited markedly lower fluorescence intensity compared to the other two cell lines (Fig 3D).

**Fig 3:**
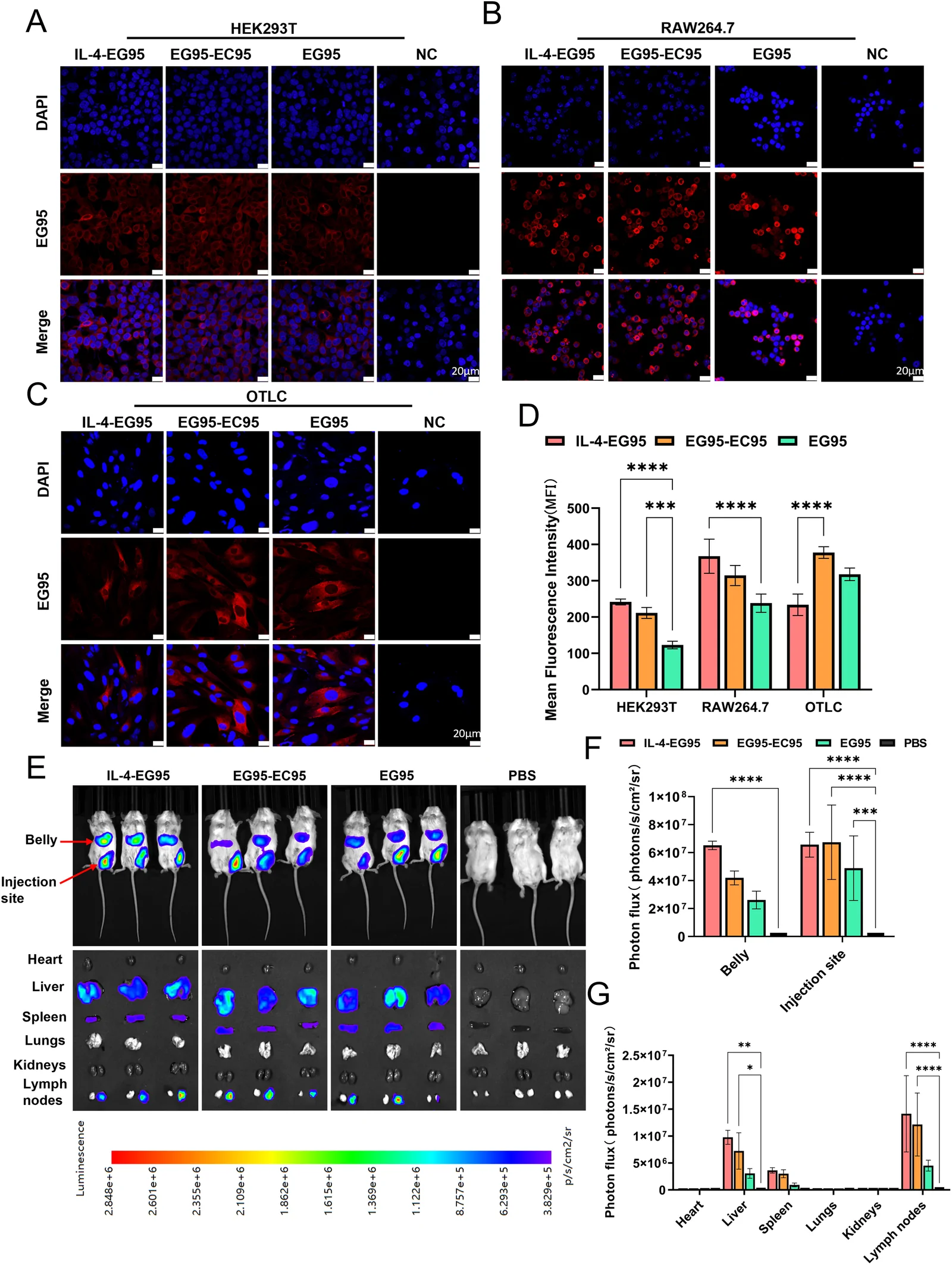
Gene delivery efficiency in vitro and biodistribution in mice of the three mRNA-LNP vaccines. At 24 hours post-treatment with the mRNA-transfection complexes, HEK293T **(A)**, RAW264.7 **(B)**, and OTLC (**C**) cells were fixed and subjected to intracellular immunofluorescence staining for EG95 protein. **(D)** Statistical analysis of mean fluorescence intensity in these cells. **(E)** In vivo bioluminescence imaging of the mice intramuscularly injected with Luciferase-tagged mRNA-LNPs, was performed 6 h post-injection under anesthesia following intraperitoneal D-luciferin administration. **(F)** Organs were harvested and imaged after euthanasia. **(G)** Signal intensity of the organs and injection site was quantified as total photon flux (photons/s/cm²/sr). Data are presented as means ± SD (n = 3), * *P* < 0.05, ** *P* < 0.01, *** *P* < 0.001, **** *P* < 0.0001.

### Biodistribution

For in vivo evaluation, luciferase-tagged target mRNA was encapsulated in LNPs and administered in mice via intramuscular injection. Six hours post-injection, D-luciferin substrate was injected intraperitoneally. In vivo imaging revealed bioluminescence signals at the injection site and within the abdominal cavity of the mice, with significantly higher radiance in the peritoneal region (Fig 3E). Ex vivo imaging of the liver and spleen further confirmed detectable bioluminescence in these organs, whereas no fluorescent signal was discernible in the heart, lungs, or kidneys. Notably, bioluminescence was also detected in the inguinal lymph nodes, demonstrating that mRNA-LNPs enable efficient translation within critical immunological organs at the early stage after injection, thereby enhancing early activation of the host immune response. These results demonstrate the three mRNA-LNPs formulations mediated efficient delivery and successful translation of target proteins in vivo.

### Safety assessment

To assess the safety of the mRNA-LNP formulation, blood biochemical parameters, including liver function (ALB, ALT, ALP, AST, TBA, T-BIL, D-BIL, and γ-GT) and renal function (UREA, CREA, UA) indicators, were evaluated in mice following mRNA-LNP vaccine intramuscular administration. The absence of notable alterations in biochemical parameters supports the favorable safety profile of all vaccine formulations (Fig 4B,4C). No clinical signs of toxicity such as hyperactivity, body rolling, pinnal reflex, convulsions, or mortality were observed during the 7-day period post-administration. Additionally, hematoxylin and eosin (H&E) staining (Fig 4A) showed no pathological alterations, including inflammatory responses or necrotic foci were noted in mice heart, liver, spleen, lung and kidney (euthanized on day 14 following an intramuscular booster immunization). Both vaccinated and PBS control groups displayed normal histology across examined tissues, featuring well-organized cardiomyocytes, absence of hepatic inflammation or edema, and preserved splenic architecture with distinct lymphoid follicles (Fig 4A).

**Fig 4:**
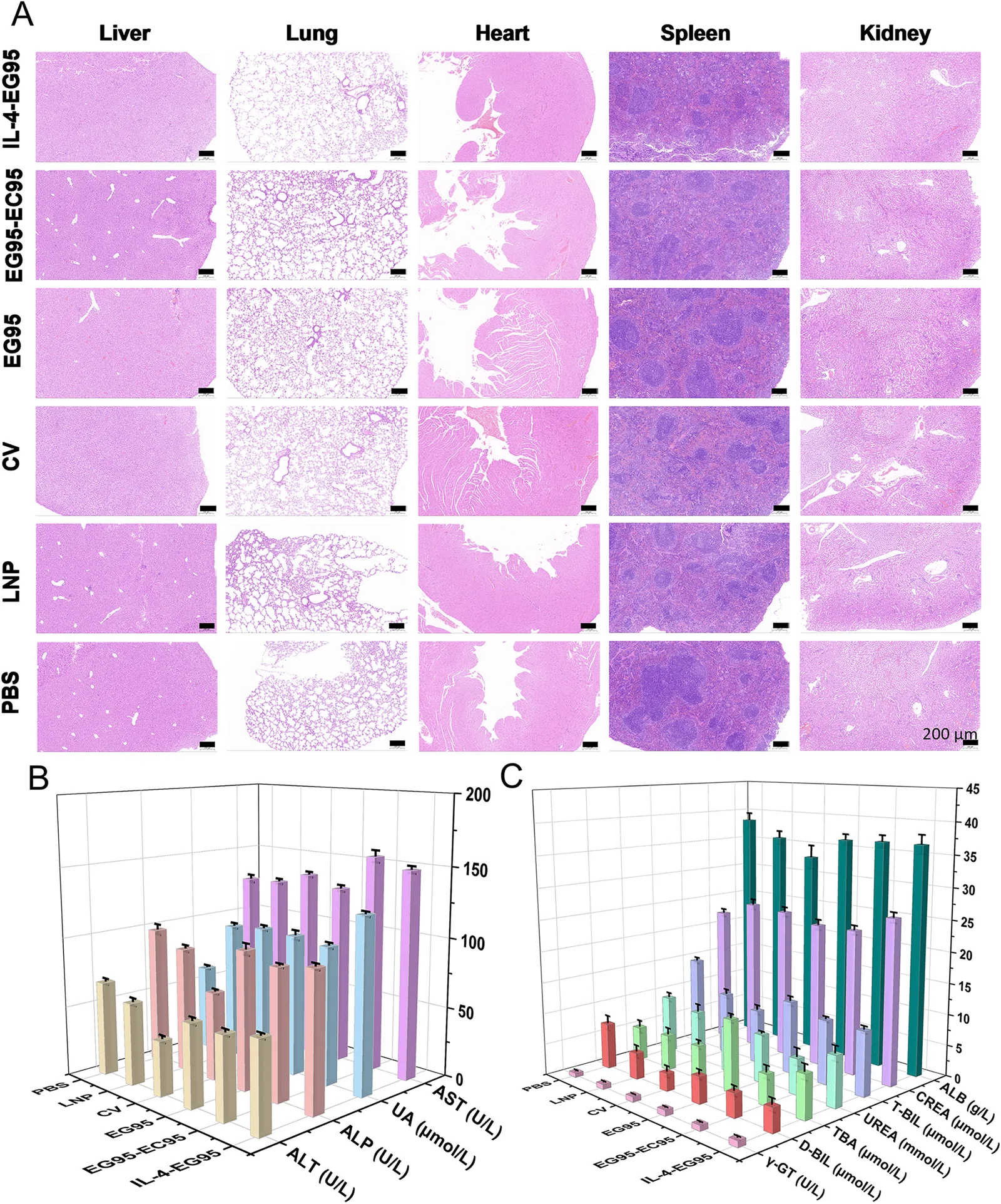
Safety profile of the three mRNA-LNPs in BALB/c mice. **(A)**. Representative H&E-stained tissue sections of heart, liver, spleen, lung, and kidney from vaccinated mice. Scale bar = 200 μm. **(B)** Serum biochemical indices of mice immunized in different groups. Serum ALT, AST, ALP and UA levels of the mice. **(C)** Serum γ-GT, D-BIL, TBA, UREA, T-BIL, CREA and ALB levels of the mice immunized in different groups.

### The mRNA-LNP vaccine elicited a humoral immune response

To assess the immunostimulatory effects of various EG95-based molecular formulations, we compared several experimental groups: EG95, EG95 in combination with EC95, and EG95 supplemented with IL-4 as a molecular adjuvant. The commercial vaccine (CV) served as a positive control, while empty LNP and PBS groups functioned as vehicle and negative controls, respectively. On day 0, mice received a primary intramuscular immunization in the left hind limb, followed by a booster injection in the right hind limb on day 14. Blood samples were collected at multiple time points post-initial immunization, specifically on days 0, 14, 28, 60, 90, and 120 (Fig 5A). Lambs were immunized intramuscularly in the neck region according to the same vaccination timeline. Similarly, a booster immunization was administered 14 days after the primary vaccination. Blood samples were collected via jugular venipuncture at 0, 14, 28, 60, 90, and 120 days following the initial immunization (Fig 5B). Anti-EG95 specific IgG, IgG1, IgG2a, IgA, and IgM antibodies were detected in vaccine immunized mice at 0, 14, 28, 60, 90, and 120 days after the primary immunization (Fig 5C), indicated the mRNA-LNP vaccines inducted a strong humoral immune response. IgM, which mediates early-stage defense against parasitic infections primarily through complement activation, decreased gradually over time in all immunized groups. Conversely, IgA, an important regulator of cell-dependent cytotoxicity exhibited a substantial increase in the mRNA-LNP vaccine-immunized groups, with a notable difference observed compared to the CV group (Fig 5C). As the predominant antibody isotype, IgG played a major role in mediating the clearance of invading pathogens [46]. In mRNA-LNP immunized Groups, IgG levels began to rise gradually from day 14, reached a peak at day 60, and subsequently showed a downward trend (Fig 5C). In contrast, the CV group showed a marked decrease in IgG levels from day 28 onward, demonstrating a significant difference compared to the mRNA -LNP immunized groups (*P* < 0.01). Furthermore, mice in the mRNA-LNP immunized groups exhibited IgG1/IgG2a ratios between 0.5 and 2.0, indicating a balanced Th1 and Th2 immune response. However, a shift toward a Th2-polarized bias was observed by day 120 (Fig 5C) [47]. Moreover, the mRNA vaccine groups demonstrated notably longer-lasting IgG antibody responses, maintaining high levels even at day 120, whereas the CV group’s antibody levels had declined to nearly baseline (Fig 5D).

**Fig 5:**
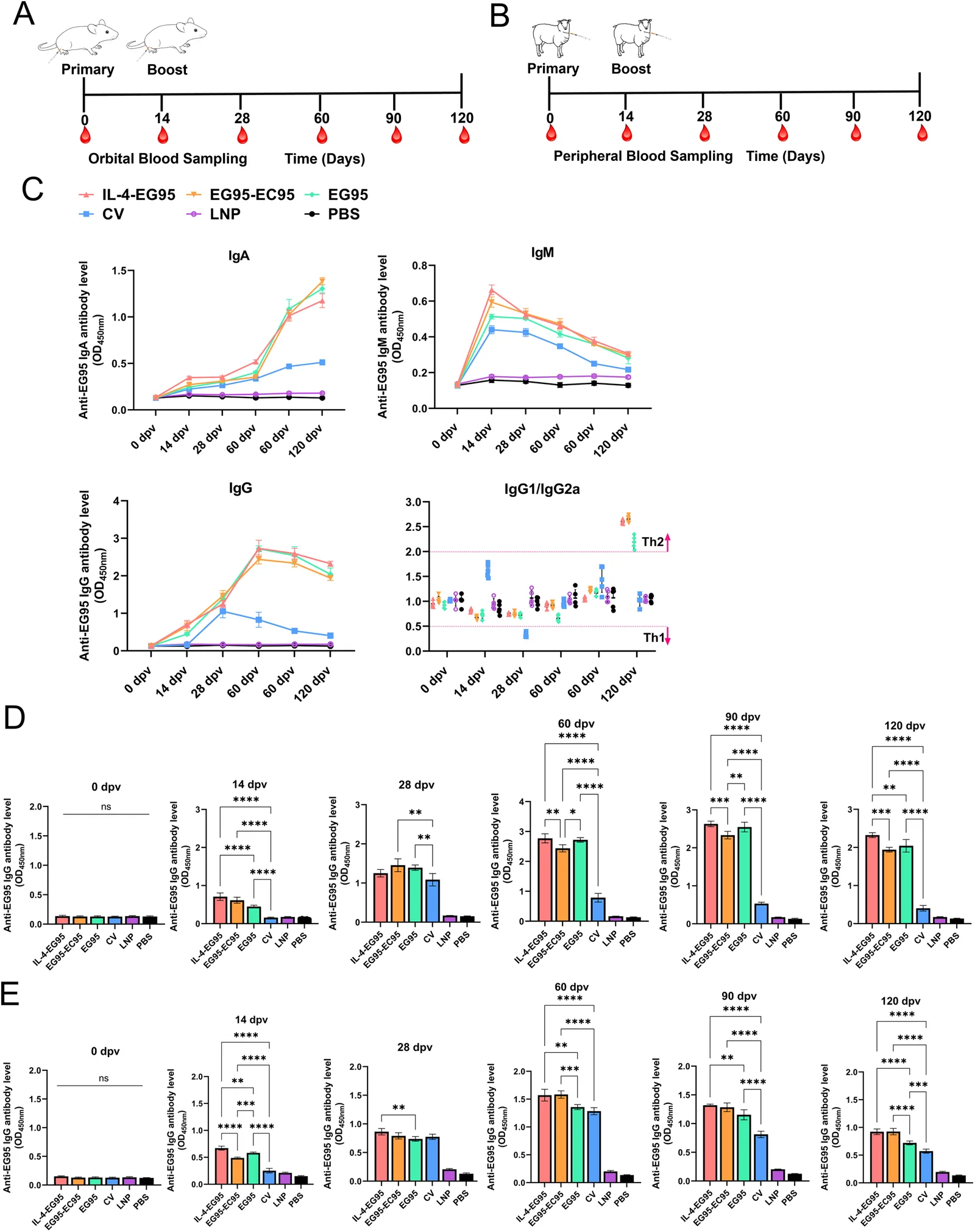
Humoral immune responses induced by the three mRNA-LNP vaccines. Schematic of the immunization protocol in BALB/c mice (**A**) and Lambs(**B**). BALB/c mice were primed in the right femoral muscle on day 0, and boosted in the left femoral muscle on day 14. Lambs were immunized via primary injection in the left cervical muscle on day 0, and booster in the right cervical muscle on day 14. Serum was collected at 0, 14, 28, 60, 90, and 120 days post-initial immunization. **(C)** Analysis of anti-EG95 IgG, IgM, and IgA antibodies in mice serum after immunization. The IgG1/IgG2a ratio was calculated as an indicator of the Th1/Th2 immune response. Statistical analysis of anti-EG95 IgG antibody levels in mice (**D**) and lamb (**E**) at different days after immunization. Data are presented as means ± SD (n = 5), * *P* < 0.05, ** *P* < 0.01, *** *P* < 0.001, **** *P* < 0.0001, ns indicates not significant (*P* > 0.05).

Following intramuscular administration of the mRNA-LNP vaccines in lambs, we conducted longitudinal monitoring of anti-EG95 IgG antibody levels. The IgG levels increased progressively with repeated immunizations and over time post-injection (Fig 5E). By day 14, a significant difference in IgG levels was observed compared to the CV group, indicating that the mRNA-LNP vaccine rapidly enhanced IgG production in sheep within a short period. Furthermore, antibody levels remained elevated at days 90 and 120, whereas a marked decline was noted in the CV group. Notably, lambs in the IL-4-EG95 and EG95-EC95 immunized groups exhibited higher IgG levels than those in the EG95 group, with a more pronounced difference observed compared to the CV group.

### The mRNA-LNP vaccine elicited a cellular immune response

Cellular immunity relies on immune cells to directly identify and eliminate infected cells and foreign pathogens [48]. Cytokines serve as critical markers for the activation of cellular immune responses and can be categorized into two functional subsets. Th1 derived cytokines (such as IL-2, TNF-α, and IFN-γ), which activate macrophages and enhance their phagocytic and cytotoxic activities particularly against intracellular pathogens. Th2 derived cytokines (including IL-4, IL-5, IL-6, and IL-13), which support B cell proliferation and differentiation, thereby mediated the initiation and maintenance of humoral immunity against extracellular pathogens. Additionally, the T/B lymphocyte proliferation assay can demonstrate whether vaccination elicits a robust immune response and effectively induces immunological memory [49]. Therefore, to evaluate cell mediated immune responses elicited by different vaccines, we assessed Th1/Th2 cytokine secretion and T/B lymphocyte proliferation on day 14 after the booster immunization. Following the booster immunization, mice immunized with the three mRNA-LNPs formulation exhibited significantly higher levels of IFN-γ compared to the CV group (Fig 6C). Notably, after IL-4-EG95 stimulation, mice splenocytes secreted greater amounts of TNF-α, IL-4, and IL-13 than those from both EG95-EC95 and EG95 mRNA-LNP immunized groups and the CV group. Furthermore, antigen specific Th2 cytokine levels were markedly elevated in the mRNA-LNP groups relative to the CV group (Fig 6C). Overall, the cytokine expression trends closely paralleled the dynamics of humoral IgG antibody responses (Fig 5D). After booster immunization, whole blood was collected from lambs via jugular venipuncture, and peripheral blood mononuclear cells (PBMCs) were isolated. Following restimulation with EG95 antigen, cytokine secretion levels were measured. Lambs in the three mRNA-LNP groups exhibited significantly higher levels of Th1 cytokines (IFN-γ and IL-2) compared to the CV group. In contrast, Th2 cytokines (IL-4 and IL-6) showed no significant difference between groups and remained at similar levels (Fig 6D). This result is directly related to the IgG antibody levels observed in sheep, as high levels of IL-4 are critical drivers of B cell proliferation and differentiation [50]. These data demonstrated that the IL-4-EG95 mRNA-LNP vaccine elicited a stronger cellular immune response compared to the commercial EG95 protein subunit vaccine.

**Fig 6:**
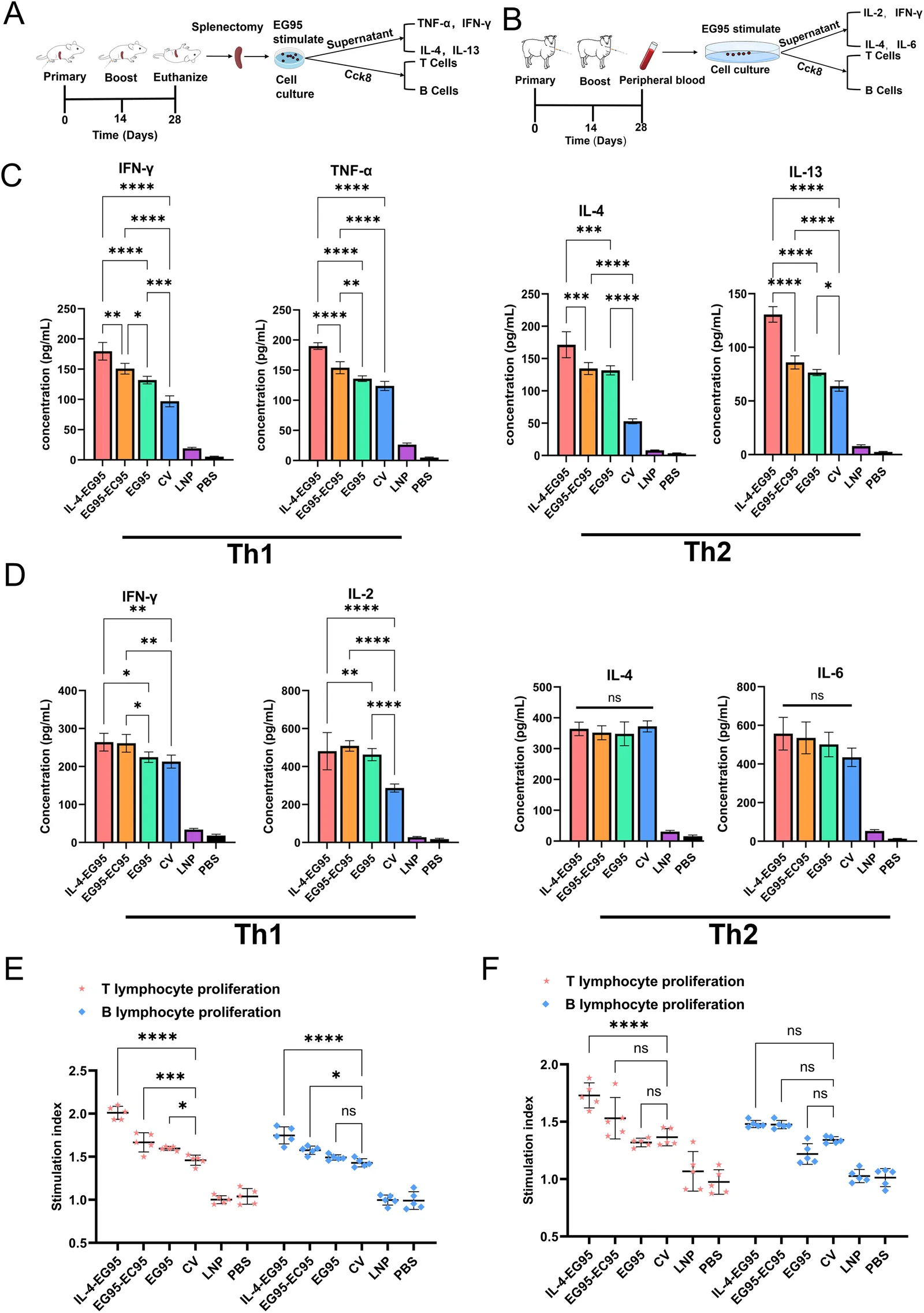
Cellular immune responses induced by the mRNA vaccines. Schematic of the immunization protocol in BALB/c mice (**A**) and Lambs **(B)**. BALB/c mice were primed in the right femoral muscle on day 0 and boosted in the left femoral muscle on day 14. Mice were euthanized on day 28 after primary immunization, and spleens were aseptically collected and processed into single-cell suspensions. Lambs were immunized via primary injection in the left cervical muscle on day 0 and booster in the right cervical muscle on day 14. Peripheral blood was collected on day 28 after primary immunization and lymphocyte isolation. Cytokine production in lymphocyte supernatants was analyzed following 72 h EG95 stimulation. Mice splenocytes (**C**) secreting Th1-derived cytokinesis (TNF-α and IFN-γ) and Th2-derived cytokines (IL-4 and IL-13), and sheep peripheral blood lymphocyte (**D**) secreting Th1-derived cytokinesis (IL-2 and IFN-γ) and Th2-derived cytokines (IL-4 and IL-6) were detected via ELISA on day 14 after booster immunization. T/B lymphocyte proliferative responses in mice (**E**) and lamb (**F**) on day 14 after booster immunization were evaluated. Data are presented as means ± SD (n = 3), *P < 0.05, ** *P* < 0.01, *** *P* < 0.001, **** *P* < 0.0001, ns indicates not significant (*P* > 0.05).

Subsequently, antigen-specific clonal expansion and proliferation of lymphocytes were evaluated, as this process directly reflects the intensity of the cellular immune response [51]. Mice in the IL-4-EG95 mRNA-LNP booster group exhibited the highest levels of both T- and B- lymphocytes proliferation, showing a significant difference compared to the CV group (*P* < 0.0001), as represented by the optimal relative proliferation index (Fig 6E). EG95-EC95 and EG95 mRNA-LNP immunized groups also demonstrated significantly greater T- lymphocytes proliferation than the CV group (*P* < 0.05). Although B- lymphocytes proliferation and clonal expansion in these two groups did not differ markedly from the CV group (Fig 6E). Following booster immunization, sheep in the IL-4-EG95 mRNA-LNP group exhibited the highest levels of T- lymphocyte proliferation in peripheral blood, showed a significant difference compared to the CV group (*P* < 0.0001). Although the other two mRNA-LNP immunized groups did not demonstrate significant differences in lymphocyte proliferation relative to the CV group (Fig 6F). No significant differences in B lymphocyte proliferation were observed among the mRNA-LNP immunization groups and CV group. These results indicated that the IL-4-EG95 mRNA-LNP vaccine effectively induced a robust cellular immune response in both mice and sheep, as evidenced by enhanced proliferation and differentiation of T- and B- lymphocytes.

Longitudinal monitoring of serum cytokine profiles revealed distinct kinetic trajectories between Th1- and Th2-type cytokines following booster immunization. The levels of IFN-γ and IL-2 rapidly increased and peaked as early as 14 days post-boost, with IFN-γ exhibited a particularly higher lever. Subsequently, both IFN-γ and IL-2 declined gradually but remained at relatively elevated levels above control group levels throughout the observation period. In contrast, IL-4 and IL-10 increased after the booster, reaching peak concentrations at 60 dpv (S3 Fig). Notably, these two Th2-type cytokines declined sharply after their peak and returned to near the control group levels thereafter.

### EG95 induced antigen-specific CD4⁺ and CD8 ⁺ T cell responses

The CD4 ⁺ T cells play a multifunctional role in adaptive immunity: they not only secrete cytokines that contribute to direct pathogen clearance but are also essential for B cell activation and proliferation [52]. Furthermore, they provide critical help for the differentiation and optimization of CD8 ⁺ T cell responses [53]. Through these mechanisms, CD4 ⁺ T cells coordinate both humoral and cellular immune pathways, thereby establishing sustained protective immunity. Notably, these functions can be quantitatively assessed by detecting antigen-specific T cell subsets, such as IFN-γ-producing CD8 ⁺ T cells (CD8 ⁺ IFN-γ⁺) and IL-4-secreting CD4 ⁺ T cells (CD4 ⁺ IL-4⁺) [54]. Therefore, on day 14 after the booster immunization, we quantified EG95-specific CD8 ⁺ IFN-γ⁺ and CD4 ⁺ IL-4 ⁺ T cells in vaccinated mice using intracellular cytokine staining (Fig 7A). The IL-4-EG95 group exhibited the highest proportions of CD4 ⁺ T cells (68.62%) (Fig 7B) and antigen-positive B cells (67.59%) (Fig 7C). All mRNA-LNP vaccinated groups showed higher percentages of these cells compared to the CV group, with significant differences relative to the PBS control group (*P* < 0.0001) (Fig 7F, 7G). These results demonstrated that the mRNA vaccines effectively induced both antigen specific humoral and cellular immune responses. Additionally, the frequencies of CD4 ⁺ IL-4⁺ and CD8 ⁺ IFN-γ ⁺ T cells were evaluated to further characterize the vaccines induced immune profiles. The mRNA-LNP groups demonstrated significantly higher proportions of CD4 ⁺ IL-4⁺ and CD8 ⁺ IFN-γ ⁺ T cells compared to the CV group (*P* < 0.0001) (Fig 7H). Notably, the IL-4-EG95 group exhibited the highest frequencies, reached 4.77% and 6.55%, respectively, with CD8 ⁺ IFN-γ ⁺ T cell levels being particularly elevated relative to all other groups. The IL-4-EG95 group exhibited a significant increase in IFN-γ ⁺ CD8 ⁺ T cells (Th1) compared to the EG95 alone group while IL-4 ⁺ CD4 ⁺ T cells (Th2) were only moderately elevated. This profile indicates a balanced Th1/Th2 response with a prominent Th1 component. These findings were consistent with the cytokine secretion profiles observed in antigen stimulated T cells (Fig 6C).

**Fig 7:**
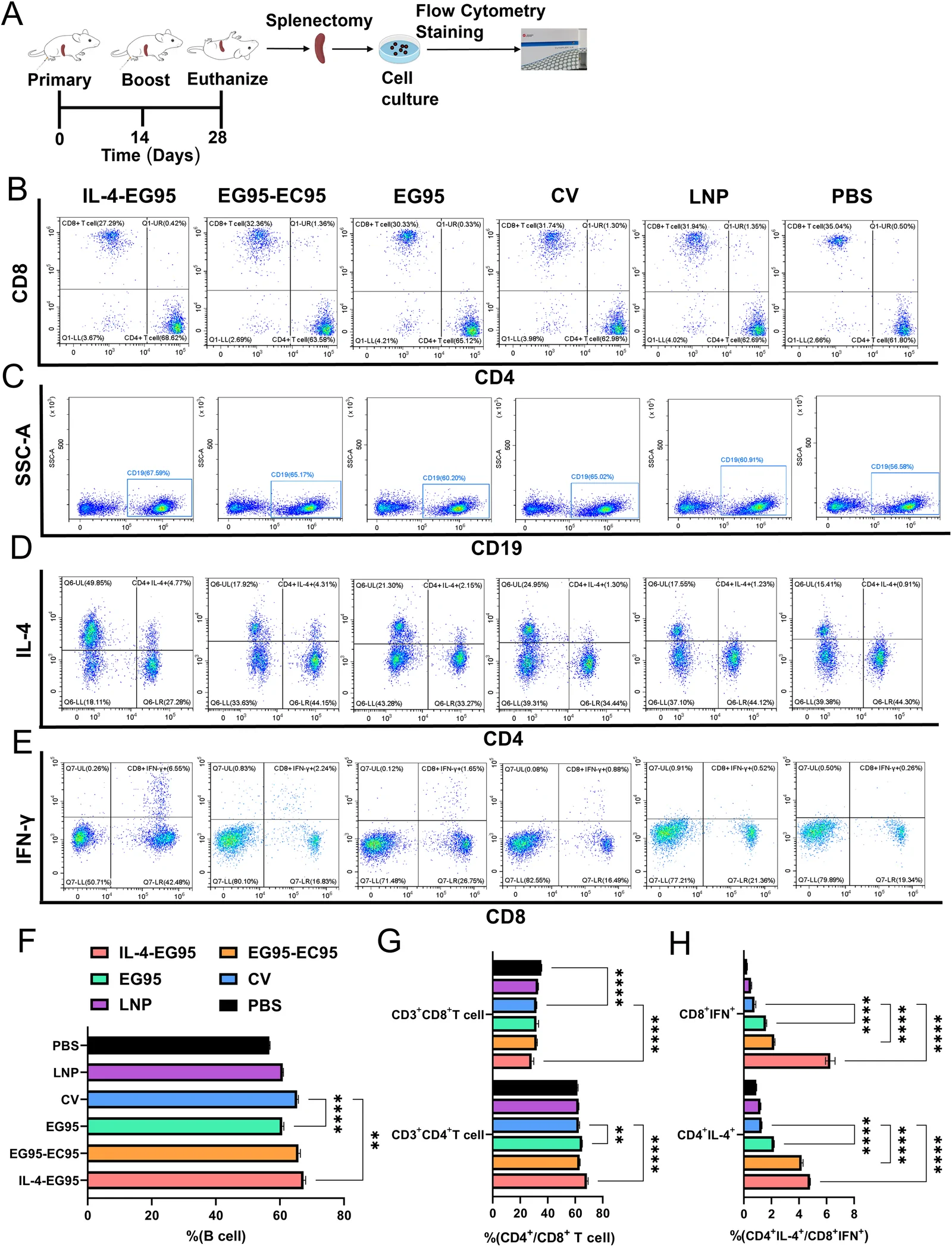
Antigen-specific lymphocyte proliferation and clonal expansion induced by various mRNA-LNP vaccines formulations. **(A)** Flowchart of antigen-specific lymphocyte preparation. Splenocytes were harvested from three mice per group on day 14 after booster immunization, followed by culture and stimulation with EG95. Representative dot plot of lymphocyte or intracellular cytokine staining is shown for each sample. Cells were immunophenotyped using anti-mouse CD3, CD4, and CD8 monoclonal antibodies to identify CD3 ⁺ CD4⁺ and CD3 ⁺ CD8 ⁺ double-positive T cells **(B)**, and anti-CD19 monoclonal antibody to detect positive B cells **(C)**. Intracellular staining for CD4/IL-4 and CD8/IFN‑γ was performed to identify CD4 ⁺ IL-4⁺ (**D**) and CD8 ⁺ IFN‑γ⁺ (**E**) double-positive cells. Statistical analysis of the percentage of positive B cells **(F)**, CD3 ⁺ CD4 ⁺ / CD3 ⁺ CD8 ⁺ positive T cells **(G)**, CD4 ⁺ IL-4 ⁺ / CD8 ⁺ IFN‑γ⁺ positive cells **(H)**. Data are presented as means ± SD (n = 3), ** *P* < 0.01, **** *P* < 0.0001.

### Protective efficacy assay

In addition to measuring key immunological parameters, such as antibody levels for humoral immunity and cytokine responses for cellular immunity, a challenge experiment is considered a critical and most direct metric for evaluating vaccine efficacy (Fig 8A) [55]. Accordingly, a challenge experiment was performed in mice 28 days post-booster immunization using *E. granulosus* protoscoleces aseptically isolated from hydatid cysts collected from sheep livers at a local slaughterhouse (S4 Fig). Six months post-challenge, all mice were necropsied to evaluate cysts development and assess the protective efficacy of the mRNA-LNP vaccines through histopathological analysis. It was evident that the number and size of *E. granulosus* cysts in the peritoneal cavity and liver of mRNA-LNP immunized mice were significantly lower than those in both the LNP and PBS groups (S5 Fig). Notably, the IL-4-EG95 group showed the highest inhibition (93%) and protection rates (91%), with some individuals achieving complete protection. In contrast, the CV group showed relatively poor protective efficacy, which is likely attributable to the low titer and short persistence of vaccine-induced protective antibodies. Furthermore, cysts in mRNA-LNP immunized mice displayed a calcified appearance and reduced viability, whereas those in the LNP and PBS groups appeared translucent and remained highly viable, with morphologically intact protoscoleces clearly observable within the cysts (Fig 8B).

**Fig 8:**
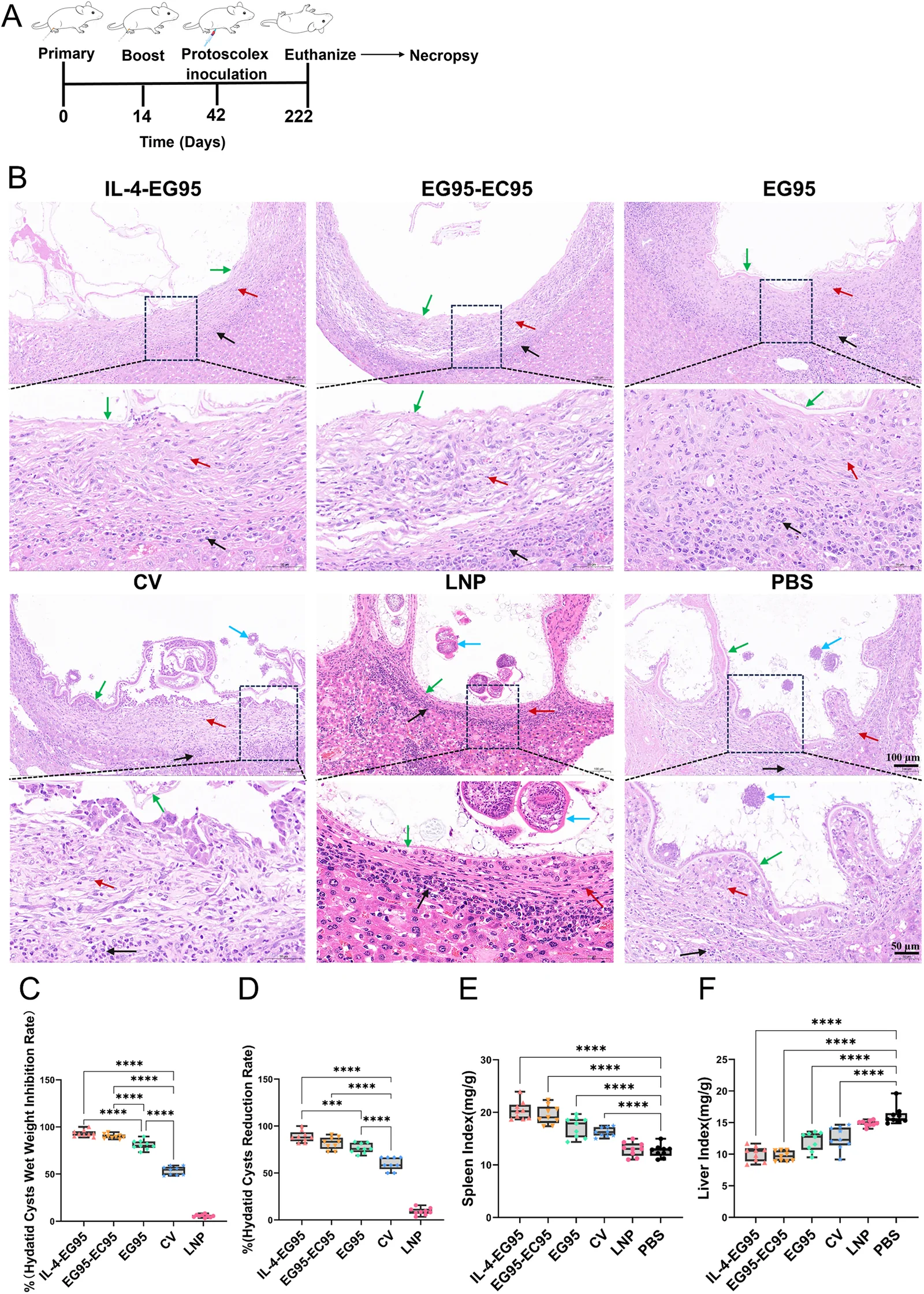
Mice were challenged intraperitoneally with protoscoleces after the booster immunization, to evaluate the protective efficacy of the mRNA vaccines. **(A)** Flowchart of the parasite challenge experiment. **(B)** Mice in different groups were infected with protoscoleces at 28 days post-booster immunization, euthanized at six months post-challenge. H&E-staining of liver tissue from all groups showed the cysts, fibrous layer, invasive inflammatory cell and liver parenchyma. Representative histopathological images of the hydatid cyst from each experimental group. Clear visualization of the cyst wall structure, including the germinal layer (GL) (green arrows). Infiltration of eosinophils (Eos) and macrophages (red arrows) in the pericystic layer, and proliferation of fibroblasts (FBs) (black arrows). Protoscolices (PSC) are visible within the cyst cavity or brood capsules in the CV, LNP, and PBS groups. Scale bar = 100 μm (main image), 50 μm (enlarged view). Statistical analysis of the cysts wet weight inhibition rate **(C)**, cysts burden inhibition rate **(D)**, liver index **(E)**, and spleen index (**F**) of different experimental groups. Data are presented as means ± SD (n = 9), ***P < 0.001, **** *P* < 0.0001.

Histopathological examination with H&E staining revealed evident cyst invasion and colonization in the liver tissues in both the CV and PBS groups, accompanied by significant inflammatory cell infiltration and resultant hepatic damage (Fig 8B). In contrast, these pathological features were observed less frequently in the mRNA-LNP immunized groups. Notably, the mRNA-LNP groups exhibited marked splenomegaly and a significantly higher spleen index compared to the PBS group (Fig 8F), indicated a robust vaccine-induced immune response characterized by extensive proliferation and expansion of effector cells [56,57]. The increase in liver index [58,59] observed in the LNP and PBS groups may be attributed to the presence of embedded cysts in the liver tissue, which can induce hepatic fibrosis and the accumulation of inflammatory cells (Fig 8E). In contrast, the mRNA vaccine groups exhibited significantly higher cyst wet weight inhibition rates (Fig 8C) and protective efficacy (Fig 8D) compared to the CV group. Notably, the IL-4-EG95 group demonstrated the strongest protection, with inhibition and protection rates reaching 93% and 91%, respectively.

### In vitro protoscolicidal activity

Following the observation that mRNA-LNPs vaccines immunization induced robust humoral and cellular immune responses in sheep, the protoscolicidal activity of the elicited antibodies was evaluated in vitro using the sheep sera (14 days after the second immunization). Following co‑incubation of sera with protoscoleces for 6 h, the 50% serum concentration group exhibited significantly higher killing efficacy compared to the 10% and 25% serum concentration groups (S6 Fig). At 12 h, marked protoscolicidal activity was observed in the 50% and 25% serum concentration groups; however, this effect was predominantly confined to the mRNA-LNPs-immunized groups, whereas no significant killing was detected in the CV group (S7 Fig). After 24 h, the mortality rate of protoscoleces in the 25% serum concentration groups from mRNA-LNPs-immunized animals exceeded 70%. And in the IL-4-EG95-immunized group, treatment with 50% serum achieved a mortality rate of 88% (S8, S10 Fig). At 48 h, the 50% serum concentration from the mRNA-LNPs-immunized group resulted in a protoscolicidal rate of 98%, while the CV group also showed a killing rate exceeding 90% (S9, S10 Fig).

## Discussion

The success of mRNA-LNP vaccines against COVID-19 has heralded a new era in vaccine development [60–62], as mRNA-based platforms offer several advantages over conventional approaches and have demonstrated promising immunogenicity in vaccines targeting influenza [63, 64] and rabies viruses [65]. Vaccination with the LEISH-F2 mRNA vaccine significantly reduced parasitic loads in the livers of *Leishmania donovani* challenged mice [35]. In a separate study, a hexavalent MDNP mRNA vaccine against *Toxoplasma gondii* conferred complete protection in immunized mice following pathogen challenge [66]. These advances in mRNA-based vaccination against parasitic diseases demonstrate the platform's substantial potential and operational feasibility. Owing to the genetic diversity and global epidemiological burden of *E. granulosus* [67–70], mRNA vaccine technology represents a highly suitable platform for the development of an effective vaccine against the parasite.

The mRNA vaccine platform enables rapid design and adjustment targeting multiple antigenic epitopes, coupled with scalable manufacturing and high cost-effectiveness [71–74]. Conventional recombinant EG95 protein vaccines against *E. granulosus* primarily rely on high levels of antibodies for parasiticidal activity [75], making robust antibody response a key criterion for an effective vaccine [76]. Therefore, we engineered an enriched version of the classic antigen EG95 by incorporating IL-4, which promotes the differentiation of T cells into the Th2 lineage and enhances the secretion of Th2 cytokines (IL-4, IL-5, IL-13). This process facilitates B cell activation and proliferation, leading to abundant production of protective antibodies that play a critical role in anti-parasitic immunity. This IL-4‑driven Th2 differentiation proceeds through both direct and indirect mechanisms: directly, IL‑4 activates STAT6/GATA3 through binds IL‑4R on naïve CD4 ⁺ T cells [77]; indirectly, it modulates antigen‑presenting cells and basophil‑T cell crosstalk, thereby establishing a microenvironment conducive to Th2 polarization [78]. Critically, IL‑4Rα/^-^mice (unresponsive to IL‑4/IL‑13) exhibit complete loss of vaccine‑induced protection against schistosomiasis, moreover passive transfer of wild‑type serum only partially restores this protective immunity protection, demonstrating that IL‑4 signaling is indispensable for antibody‑mediated Th2 immunity [79]. A flexible linker was used to fuse IL-4 with EG95, ensuring proper folding and presentation of antigenic epitopes. In our study in silico structural modeling using AlphaFold2 and PyMOL confirmed that the linker preserves the conformational integrity of both T-cell and B-cell epitopes within EG95, prevents epitope distortion or migration, and supports correct folding and surface exposure of all fused domains. Collectively, these features ensure independent domain refolding and maintain epitope authenticity—key determinants of enhanced immunogenicity. The experimental results closely aligned with our hypotheses, demonstrating a strong humoral immune response (Fig 5D) as well as potent cellular immunity (Fig 6C). Given the genetic diversity and worldwide distribution of *E. granulosus*, a multi-antigen combined immunization strategy represents one of the most promising approaches for achieving broad protective immunity. The mRNA vaccine platform is particularly well-suited for this purpose, as it allows flexible and efficient design and assembly of complex molecular constructs with straightforward production processes [80,81]. In this study, we designed a fusion antigen by linking the immunodominant antigen EG95 (from genotype G1) and the immunogenic antigen EC95 (from genotype G6) via a flexible linker to ensure proper epitope presentation. The EG95 vaccine antigen, originally derived from *Echinococcus granulosus* sensu stricto (G1 genotype), confers high protection against homologous challenge [82]. However, the *E. granulosus* sensu lato complex includes multiple genotypic variants, notably *E. canadensis* (G6/G7 genotypes), which is responsible for a substantial proportion of human cystic echinococcosis in many regions [83]. The inability of G6-genotype EG95-related proteins (EG95-1G6 and EG95-5G6) to bind antibodies raised by EG95-1G1 vaccination in sheep is likely attributable to amino acid differences in the encoded FnIII domain, which may alter epitope conformation [82]. To address this, we designed an EG95-EC95 multivalent mRNA-LNP vaccine that incorporates both antigens, aiming to induce antibodies targeting conserved and genotype-specific epitopes. This approach is particularly valuable in areas where both species are co-endemic, such as parts of North Africa, the Middle East, and Central Asia [83]. Our findings demonstrate that this bivalent formulation induces robust immune responses, supporting its potential as a broadly protective vaccine against the *E. granulosus* sensu lato complex. Immunization results demonstrated that the EG95-EC95 mRNA vaccine not only induced strong antigen-specific humoral immune responses (Fig 5D) but also stimulated significantly elevated levels of cytokine secretion, indicating robust cellular immunity (Fig 6C). This study provides an experimental foundation for multi-antigen combination vaccines and demonstrates the feasibility of using mRNA technology to develop multivalent vaccines against complex parasitic pathogens. It is evident that the two engineered molecular constructs based on EG95 elicited stronger immune responses compared to the unmodified EG95 antigen, indicated that our molecular modification strategy successfully enhanced its immunogenicity. Although the immune effect of the single-molecule EG95 mRNA vaccine was slightly lower than that of the modified versions, it still outperformed the commercially available vaccine. The EG95-based mRNA vaccine demonstrated significant immunogenic advantages, particularly in inducing high levels of protective antibodies with prolonged durability. Notably, antigen-specific antibodies remained at elevated levels even 12 months after booster immunization (S11 Fig).

IgM is the earliest-induced adaptive antibody following infection [84], owing to its ability to enhance antigen-specific immune responses and restrict pathogen dissemination [85], it is considered a bridging antibody that facilitates the transition from innate to adaptive immunity [86,87]. In chronically infected mice, the primary antibodies produced against the antigen are IgG3 and IgM [88] stimulated by T cell-independent mechanisms [89]. In our study, IgM levels in all vaccine-immunized groups exhibited a gradual decline over time. On day 14 after the booster immunization, the IL-4-EG95 group showed IgM antibody titers 1.5-fold higher than those in the CV group. By day 120, IgM levels in all groups had reduced to nearly undetectable. Meanwhile, the kinetic profile of IgA antibodies resembled that of IgG, with one notable distinction: the CV group exhibited only slow and limited elevation in IgA, whereas the mRNA vaccine-immunized groups induced robust and sustained IgA antibody production (Fig 5C).

An IgG1/IgG2a ratio of ≤0.5 is indicative of a Th1 polarized immune response, while a ratio ≥2.0 suggests Th2 polarization [47], ratios between 0.5 and 2.0 reflect a mixed or balanced Th1/Th2 profile [90,91]. Th1 type cytokines generally promote pro-inflammatory reactions, contributing to the elimination of intracellular parasites and potentially exacerbating autoimmune pathologies. In contrast, Th2 type cytokines including interleukin IL-4, IL-5, IL-10, and IL-13 are associated with anti-inflammatory functions [92]. Thus, the Th1/Th2 cytokine secretion profile may serve as a reflection of the host’s immunoregulatory balance [93,94]. A predominant Th1 response is linked to pro-inflammatory autoimmune disorders such as type 1 diabetes and rheumatoid arthritis, whereas a Th2-polarized response may predispose the host to allergic conditions. Consequently, an effective vaccine should ideally elicit a balanced Th1 and Th2 response to ensure appropriate immunological adaptation. In this study, the mRNA-LNP immunized groups exhibited IgG1/IgG2a ratios between 0.5 and 2.0 from the initial immunization through day 90, indicative of a mixed or balanced Th1/Th2 response. Although IL-4 is classically associated with Th2 responses, its incorporation as a molecular adjuvant in this study did not induce pathological Th2 skewing. Instead, longitudinal monitoring demonstrated a durable IFN-γ-producing T cell response and sustained IgG2a antibody levels. Our data demonstrate that the IL-4 fusion provides synergistic rather than merely additive enhancement of antigen specific immunity, as evidenced by significantly elevated antigen‑specific antibody titers compared with the antigen alone. The functional significance of direct physical linkage between cytokine and antigen has been confirmed [95]. By covalently tethering IL-4 to the antigen, the fusion construct ensures their concurrent delivery to the same antigen-presenting cells, thereby enabling precise spatiotemporal coordination of cytokine signaling and antigen processing—a key advantage over systemic co-administration. This finding indicates that IL-4 fusion elicits a balanced Th1/Th2 response, without deviating toward a pathological Th2-biased phenotype, thereby supporting protective immunity against cystic echinococcosis (CE) [18,96]. Furthermore, the principle of cytokine–antigen fusion–mediated synergy has been independently validated in multiple parasitic vaccine platforms [97]. The CV group maintained a mixed Th1/Th2 response throughout the observation period (Fig 5C).

Vaccine efficacy relies critically on potent cellular and high-titer humoral immune responses [98,99]. This study demonstrated that all three mRNA vaccines induced superior humoral and cellular immunity compared to a commercially available vaccine, eliciting high levels of specific protective antibodies characterized by both elevated titers and prolonged durability. Significant increases were observed in both Th1-type (IFN-γ and TNF-α) and Th2-type (IL-4 and IL-13) cytokines (Fig 6C). Furthermore, T and B-cell proliferation assays confirmed the generation of antigen-specific effector and memory T and B cells following mRNA vaccination (Fig 6E). Additionally, a significantly increased frequency of CD4 ⁺ IL-4 ⁺ T cells and CD8 ⁺ IFN-γ ⁺ T cells was detected (Fig 7H), indicating that the successful induction of a potent humoral immune response involved coordinated antibody production facilitated by T helper cells, along with the activation of cytotoxic T lymphocytes [100].

Challenge experiments represent a critical indicator for evaluating the efficacy and practical applicability of a vaccine [101,102]. Although all three mRNA vaccines developed in this study were capable of inducing robust humoral and cellular immune responses in animals, the results of the challenge experiment provide a more direct and objective assessment of vaccine efficacy and utility. Therefore, we conducted a parasite infection challenge trial. The results revealed that all the three mRNA-LNP vaccines, especially the IL-4-EG95 mRNA-LNP vaccine, demonstrated a marked inhibitory effect on both the establishment and growth of hydatid cysts, as well as the formation of protoscoleces in the cysts. In contrast, the commercial vaccine afforded weaker protection and limited scolicidal effects. Collectively, these findings support the potential real-world applicability of the mRNA vaccine platform.

The in vitro protoscolicidal activity of sera from immunized sheep provides important evidence for the functional quality of antibodies elicited by the mRNA-LNP vaccine.The early emergence of protoscolex killing activity in mRNA-LNP immunized groups at 6 h, particularly at higher serum concentrations, indicated that these vaccines induced functional antibodies with sufficient avidity to mediate parasite clearance. Notably, the absence of significant protoscolex killing in the conventional vaccine (CV) group at 6 h highlights the superiority of the mRNA-LNP-induced humoral response, which may attributable to differences in antibody isotype, epitope specificity, or Fc-mediated effector functions. Although both mRNA and CV vaccines ultimately achieved high protoscolex killing rates at 48 h, the accelerated onset of functional immunity efficacy by the mRNA-LNP formulation showed distinct translational relevance. The findings are consistent with those reported by Woollard et al. [103,104], that also demonstrated in vitro parasiticidal activity mediated by antibodies from EG95‑immunised sheep. In cystic echinococcosis, where rapid immune intervention may be critical to prevent cyst establishment or dissemination, a vaccine capable of eliciting prompt functional antibody responses offers a clinically meaningful advantage. Lightowlers and Heath have comprehensively reviewed evidence demonstrating that vaccination in sheep EG95 induces complement‑fixing antibodies associated with robust anti-parasitic protection. And in the majority of vaccinated animals, no hydatid cysts develop following experimental challenge [9]. The mRNA-LNP platform demonstrated robust and rapidly inducible efficacy, achieved near-complete protoscolex killing by 48 hours, underscores its potential as a next-generation vaccine strategy against echinococcosis. These findings support advancing mRNA-LNP platforms for parasitic diseases (S10, S12 Fig), where in the quality and kinetics of the humoral immune response are key determinants of protective efficacy. Nevertheless, a comprehensive validation of vaccine efficacy against *E. granulosus* requires further evaluation in sheep-the primary intermediate host of the parasite. The further studies would involve the use of infectious eggs derived from definitive hosts (dogs) and include an evaluation of the magnitude of the vaccine-induced immune response in sheep. These investigations will be addressed in subsequent studies.

Manufacturing scalability, GMP compatibility, production costs and scale-up feasibility are the key issues to be considered in the development of mRNA vaccines. The development of indigenous mRNA platforms has substantially advanced the localized manufacturing of LNP lipids (e.g., the SM-102 equivalent) [105]. It provide the basis for the manufacturing scalability of our mRNA vaccine. Besides, mRNA production is synthetic, cell‑free, and platform‑based, inherently GMP‑compatible. WHO and other agencies have established regulatory frameworks for mRNA vaccines, and decentralized modular manufacturing (e.g., IVI’s DeCAFx) is already validated. The cost of vaccines is also the key to restricting the application of vaccines. Technoeconomic analyses show mRNA vaccines can achieve ~ €0.88–$2 per dose, competitive with or lower than recombinant protein vaccines [106]. With ongoing technological advancements, mRNA vaccines are increasingly demonstrating cost-efficiency advantages. Moreover, the Rabies mRNA vaccine (CTN-1 strain) has entered clinical trials [107], which makes the high scale-up feasibility and application prospects of animal mRNA vaccines. Our mRNA vaccines will continue to be optimized and offering a promising strategy for controlling *E. granulosus* sensu lato transmission.

## Materials and methods

### mRNA production and LNP encapsulation

The amino acid sequences of the protective antigen EG95 (G1 sheep strain; GenBank: CAA62433.1) and EC95 (G6 camel strain; GenBank: AH013645.2) were used as references. Signal peptide and transmembrane domain predictions were performed using SignalP 5.0 and TMHMM 2.0 [108], respectively. Three antigen constructs were designed, IL-4-EG95 (aa 16–132), EG95-EC95 (aa 16–132) and EG95. To enhance the stability and bioactivity of the fusion proteins, flexible linkers were incorporated between EG95 and IL-4 (GenBank: P07750.1), as well as between EG95 and EC95. Their positions within the tertiary structure of the EG95 protein were predicted using AlphaFold 2 and visualized with PyMOL [109]. An IL-2 signal peptide (GenBank: AAB86861.1, aa 1–20) was fused to the N-terminus of each construct to facilitate protein secretion [110], to enhance antigen uptake efficiency. mRNA was synthesized via in vitro transcription using T7 RNA polymerase and a linearized plasmid DNA template optimized with codon usage for EG95.

For LNP formulation, lipids [111–113] including an ionizable lipid, 1,2-distearoyl-sn-glycero-3-phosphocholine (DSPC), cholesterol, and a PEGylated lipid were dissolved in ethanol. The lipid mixture was mixed with mRNA dissolved in 10 mM citrate buffer (pH 4.0) at a 1:3 volumetric ratio using a microfluidic mixer. The resulting LNPs were dialyzed against phosphate-buffered saline (PBS, pH 7.4) and concentrated using Amicon Ultra centrifugal filters (Millipore, USA) with a molecular weight cutoff of 10 kDa. Particle size and zeta potential were characterized by dynamic light scattering (DLS) using a Zetasizer Nano ZS (Malvern Instruments, UK). Encapsulation efficiency was measured via fluorescence staining, in which unencapsulated cargo was quantified following dye addition. For morphological analysis using transmission electron microscopy (TEM), both empty and mRNA-loaded LNPs were adsorbed onto 200-mesh copper grids (EMCN, China). The samples were then negatively stained and examined under a JEM-1400Flash transmission electron microscope (Hitachi, Japan) operating at 80 kV.

### In vitro study

#### Cell culture.

The human embryonic kidney cell HEK293T (ATCC CRL-3216), the murine macrophage-like cell RAW 264.7 (ATCC TIB-71), and sheep testicular stromal cells (OTLC, IMMOCELL, China/NICR 4201PAT-CCTCC01465, China) were cultured in Dulbecco’s Modified Eagle Medium (DMEM; Gibco) supplemented with 10% fetal bovine serum (FBS; Gibco) at 37°C in a 5% CO_2_ incubator (Thermo Fisher Scientific, USA).

#### Cell transfection study.

To evaluate the expression of mRNA constructs, HEK293T, RAW264.7, and OTLC cells were seeded in 12-well plates at a density of 2 × 10^5^ cells per well and cultured in DMEM supplemented with 10% (v/v) fetal bovine serum (FBS), 100 μg/mL streptomycin, and 100 U/mL penicillin (Solarbio, China) at 37°C in a 5% CO_2_ atmosphere. After 8 hours of incubation, cells were transfected with three mRNA complexes (1 µg per well) using CALNP mRNA transfection reagent (D-nano, China) for 24 h and 48 h in vitro, respectively. Untreated cells served as the control group.

#### Real-time RT-PCR (RT-qPCR).

Transgene expression was analyzed by quantitative reverse transcription PCR (RT-qPCR) following the incubation period. Briefly, harvested cells were centrifuged and the supernatant was discarded. Total RNA was isolated using TRIzol reagent (Invitrogen, USA) according to the manufacturer's protocol. First-strand cDNA was synthesized using the HiScript II 1st Strand cDNA Synthesis Kit (Vazyme, China). Quantitative PCR was performed using TB Green Premix Ex Taq II (Takara, Japan) on a CFX96 Touch Real-Time PCR Detection System (Bio-Rad, USA). The specificity and size of the qPCR amplicons were verified by agarose gel electrophoresis, followed by imaging with a ChemiDoc XRS system (Bio-Rad, USA). The primers sequences are as EG95-Forward: 5’-GAGACAAGGACCACCGAGAC and EG95-Reverse: 5’-AGGGGTTCACTGCTT TCAGG. Glyceraldehyde‑3‑phosphate dehydrogenase (GAPDH) was used as the endogenous control gene, and the relative mRNA expression levels were determined using the 2^-ΔCtΔCt^ method.

### Western Blot (WB)

To quantitatively analyze protein expression, Western blotting was performed to detect the target protein EG95 in different cell types and culture supernatants after transfection treatment. Briefly, harvested cells were lysed on ice using RIPA buffer (Beyotime, China) supplemented with a protease inhibitor cocktail (1:100) and PMSF (Thermo Fisher Scientific, USA). After 40 minutes of lysis, loading buffer was added to both the lysates and collected culture supernatants. The samples were then boiled for 10 minutes and subjected to gel electrophoresis. Proteins were electrophoretically transferred from SDS-PAGE gels to PVDF (Thermo Fisher Scientific, USA) membranes. The membranes were blocked with 5% non-fat milk (Beyotime, China) for 2 h at room temperature and subsequently incubated with an anti-EG95 mouse monoclonal antibody (1:500 dilution, generated in-house) overnight at 4°C. Following three washes with TBST, the membranes were probed with a horseradish peroxidase (HRP) conjugated goat anti-mouse antibody (1:5000 dilution, Abcam, UK) for 50 min at room temperature. After additional TBST washes, protein bands were detected using ECL ultrasensitive chemiluminescent (Thermo Fisher Scientific, USA) substrate and imaged with a ChemiDoc XRS system (Bio-Rad, USA). β-tubulin served as the loading control for normalization, untreated cells were used as the control.

### Indirect immunofluorescence (IF) assay

Indirect immunofluorescence was performed using anti-EG95 monoclonal antibody (produced and stored in-house) and goat anti-mouse IgG (H + L) cross-adsorbed antibody (Thermo Fisher Scientific, USA). Briefly, HEK293T, RAW264.7, and OTLC cells were seeded on glass-bottom cell culture dishes (Thermo Fisher Scientific, USA) at a density of 1 × 10^5^ cells per well and incubated at 37°C for 24 h. The cells were transfected with the three different mRNA (1 μg per well) encapsulated in LNP for 24 hours, respectively. Untreated cells were used as the control. Then, cells were washed with PBS (pH 7.4) and fixed with 4% paraformaldehyde for 30 min at room temperature, followed by permeabilization with 1% Triton X-100 (Beyotime, China) at 37°C for 1 h. The cells were then incubated with 100 μL of primary anti-EG95 monoclonal antibody (1:500 dilution) at 37°C for 2 h. After thorough washing, the cells were incubated with 100 μL of goat anti-mouse IgG(H + L) Highly Cross-Adsorbed Antibody, Alexa Fluor Plus 594 1:1000 dilution (Invitrogen, USA) at 37°C for 1.5 h in a humidified chamber. Cell nuclei were stained with DAPI (Abcam, UK) at 37°C for 10 min. Images were acquired using a laser-scanning confocal microscope (Leica SP8, Germany).

### In vivo study

#### Animals.

Female BALB/c mice (6–8 weeks old) and lambs (4–6 months old) used in this study were obtained from the Lanzhou Veterinary Research Institute, Chinese Academy of Agricultural Sciences (Lanzhou, China). All animal experiments were conducted in accordance with protocols approved by the Institutional Animal Care and Ethics Committee of the Lanzhou Veterinary Research Institute, Chinese Academy of Agricultural Sciences (Approval No.: LVRIAEC-2024–009).

### Biodistribution study

BALB/c mice were intramuscularly injected with LNPs encapsulating 5 µg of luciferase (Fluc)-tagged target mRNA. PBS (pH 7.4)-injected mice served as the negative control. Six hours post-injection, the mice were administered intraperitoneally with 3 mg of D-luciferin (MCE, USA) dissolved in aseptic PBS (pH 7.4). After luciferin injection 10 minutes, the mice were anesthetized using isoflurane (RWD, China). Bioluminescence imaging was conducted using an IVIS Spectrum imaging system (PerkinElmer, USA). After imaging, the mice were euthanized, and major organs (liver, spleen, heart, lungs, kidneys, and inguinal lymph nodes) were harvested for ex vivo imaging. Signal intensity was quantified as total photon flux (photons/s/cm^2^/sr).

### Immunization scheme and safety evaluation of mRNA- LNP in mice

Ninety 6-week-old female BALB/c mice were randomly divided into six groups (n = 15 per group). Three groups were immunized via intramuscular injection in the thigh with 20 µg of mRNA-LNP vaccine per mouse, including IL-4-EG95 mRNA-LNP group, EG95-EC95 mRNA-LNP group and EG95 mRNA-LNP group. The remaining animals were assigned to three control groups: a positive control group (CV) administered a commercial vaccine (Chongqing Auleon Biologicals Co., Ltd.) 20 ug per mouse, and two control groups administered either PBS (pH 7.4) or the LNP formulation to serve as the negative and vehicle controls, respectively, via the same intramuscular route. A booster immunization was administered on day 14 post-vaccination (dpv). Blood samples were collected from the orbital venous plexus at scheduled time points (0, 14, 28, 60, 90, and 120 dpv), and serum was separated by centrifugation at 4000 rpm for 15 minutes at 4°C.

Three BALB/c mice in each group were euthanized two weeks after a booster immunization administered intramuscularly in the hind limb muscles with mRNA-LNP. Major organs, including the heart, liver, spleen, lung, and kidney, were harvested and fixed in 4% paraformaldehyde (Beyotime, China) for 72 hours. Following dehydration through a graded ethanol series, the tissues were embedded in paraffin. Sections of 4 μm thickness of the tissues were prepared and stained with hematoxylin and eosin (H&E). Histopathological images were captured using an optical microscope (Olympus, Japan) for further evaluation. Biochemical parameters were analyzed using a Chemray-240 fully automated biochemistry analyzer (Rayto, China).

### Sheep vaccination experiments

Forty-two 4-month-old lambs were randomly allocated into six groups (n = 7 per group). Lambs in the three immunized groups received an intramuscular injection of the mRNA-LNP vaccine (100 μg per animal) in the neck region, including IL-4-EG95 mRNA-LNP group, EG95-EC95 mRNA-LNP group and EG95 mRNA-LNP group. Three The control groups were administered as follows: the commercial *Echinococcus* vaccine (CV, 100 μg per animal) served as the positive control, while LNP (100 μg per animal) and PBS (pH 7.4) served as vehicle and negative controls, respectively. A booster immunization was administered 14 days after the primary vaccination. Peripheral blood samples were collected at specified time points (0, 14, 28, 60, 90, and 120 dpv), and serum was isolated for subsequent analysis.

### EG95-specific IgG, IgM, IgA, IgG1 and IgG2a antibodies in peripheral blood of mice

Levels of anti-EG95 IgG, IgM, IgA, IgG1, and IgG2a in mice serum were quantified by enzyme-linked immunosorbent assay (ELISA). Briefly, 96-well polystyrene plates were coated with purified yeast-expressed recombinant EG95 antigen (200 μL/well, 10 μg/mL), and incubated at 37°C for 2 hours followed by overnight at 4°C. After three washes with PBS containing 0.1% Tween-20 (PBST), the plates were blocked with 5% bovine serum albumin (BSA; Sigma-Aldrich, USA) in PBS (200 μL/well) at 37°C for 2 hours, followed by three additional washes with PBST. Serum samples were diluted 100-fold and added to the antigen-coated plates, followed by incubation at 37°C for 30 min. After washing, the plates were incubated with HRP-conjugated goat anti-mouse IgG, IgM, IgA, IgG1, and IgG2a antibodies (Abcam, UK) for 30 min at 37°C, respectively. Following another washing step, color development was initiated by adding 3,3′,5,5′-tetramethylbenzidine (TMB; Surmodics, USA) substrate. The reaction was terminated with 1 M H_2_SO_4_, and the optical density (OD) at 450 nm was measured using a microplate reader (Bio-Rad, USA).

### Detection of EG95-specific IgG antibodies in lamb peripheral blood

EG95-specific IgG antibodies in collected lamb serum samples were quantified using an enzyme-linked immunosorbent assay (ELISA). Briefly, 96-well plates were prepared as described in previous sections. All serum samples were diluted 1:100 in PBS (pH 7.4) and added to the antigen-coated plates, followed by incubated at 37°C for 30 minutes. After washing, HRP-conjugated donkey anti-sheep IgG antibody (Abcam, UK) was added at a dilution of 1:50,000, followed by incubation at 37°C for 30 minutes and three additional washes. Color development was initiated by adding TMB substrate and incubating at 37°C for 15 minutes. The reaction was stopped with 1 M H_2_SO_4_, and the OD at 450 nm was measured using a microplate reader (Bio-Rad, USA).

### Flow cytometry

Spleens of 3 mice in each group were harvested at 28 dpv and mechanically dissociated in cold PBS. The cell suspension was filtered through a sterile 70-μm nylon mesh. Erythrocytes were lysed using Tris-ammonium chloride buffer (Sigma-Aldrich, USA), and the reaction was quenched with PBS. The resulting cells were pelleted and resuspended at a concentration of 1 × 10⁶ cells per tube in 1.5 mL centrifuge tubes. Subsequently, cells were resuspended in 300 µL of flow cytometry staining buffer and stained with the following fluorescently conjugated antibodies for 35 minutes at room temperature in the dark, including Brilliant Violet 421 anti-mouse CD3ε (BioLegend, USA), FITC anti-mouse CD4 (BioLegend, USA), APC anti-mouse CD8α (BioLegend, USA), and PE anti-mouse CD19 (BioLegend, USA). After staining, the cells were centrifuged, washed twice with PBS, and resuspended in 500 µL of fixation buffer. Flow cytometry was performed on a CytoFLEX LX instrument (Beckman Coulter, USA). CD4 ⁺ T cells, CD8 ⁺ T cells, and B cells were quantified per 1 × 10^5^ acquired events.

### Intracellular cytokine staining

Splenocytes (5 × 10^6^ cells per sample) prepared as described above were stimulated with EG95 peptide (10 μg/mL) for 1 hour at 37°C. Brefeldin A (BFA; Thermo Fisher Scientific, USA) was then added at a concentration of 1 μL per sample to inhibit intracellular protein transport during the final 4 hours of stimulation. After incubation, cells were washed and stained with anti-mouse CD4-FITC and anti-mouse CD8α-PE for 30 minutes at 4°C, respectively. Subsequently, the cells were fixed, permeabilized, and intracellularly stained with anti-mouse IFN-γ-Brilliant Violet 421 (BioLegend, USA) and anti-mouse IL-4-PE/Cyanine7 (BioLegend, USA) for 30 minutes at 4°C in the dark, respectively. Finally, the cells were washed and resuspended in fixation buffer for flow cytometric analysis.

### The proliferation of lymphocytes

Mouse splenic lymphocytes were prepared as previously described. Lamb peripheral blood was collected into anticoagulant vacuum tubes (containing sodium citrate as the anticoagulant) and transported to the laboratory. Lamb peripheral blood lymphocytes were isolated using Ovine Peripheral Blood Lymphocyte Separation Medium (TBD, China). Lymphocyte suspensions were adjusted to 5 × 10^6^ cells/mL in RPMI 1640 medium (Gibco, USA) with 10% fetal bovine serum, seeded into a 96-well plate (100 µL per well), and stimulated with 10 µg/mL of EG95 protein (produced in-house by fermentation in Pichia pastoris), and T- and B-lymphocyte proliferation was assessed using Con A (5 μg/mL) and LPS (20 μg/mL) [114,115], respectively. Unstimulated lymphocytes served as the negative control.

After 72 h of incubation at 37°C in a 5% CO_2_, the cell culture supernatant was collected for cytokine detection and replaced with fresh medium. Then, 10% CCK-8 solution (Beyotime, China) was added to each well, followed by incubation at 37°C for 1 h. The absorbance at 450 nm (OD_450_) was measured using a microplate reader. The stimulation index (SI) was calculated as follows: SI = (EG95 stimulated well OD_450_ value – Blank OD_450_ value) / (Control OD_450_ value– Blank OD_450_ value) × 100% [116].

### Cytokine expression from splenocytes

As described above, the mouse splenic lymphocytes were seeded in a 96-well plate (5 × 10^6^/per well, 6 wells) and stimulated with EG95 protein for 72 hours. Subsequently, all culture supernatants were collected, and cytokine levels—including IFN-γ, TNF-α, IL-13, and IL-4 were measured using ELISA kits (Elabscience, China).

Lamb lymphocytes were seeded in a 96-well plate (5 × 10^6^/per well, 6 wells) and stimulated with EG95 protein (10 µg/mL) for 72 hours. Subsequently, all culture supernatants were collected, and cytokine levels, including IFN-γ, IL-2, IL-4 and IL-6 were measured using ELISA kits (Solarbio, China).

### Cytokine dynamics

The dynamic cytokine profiles in mice following mRNA-LNP immunization were continuously monitored. The levels of cytokines, including IL-2, IFN-γ, IL-4, and IL-10, in serum were detected at 0, 28, 60, 90, and 180 days post-vaccination (dpv) using commercial cytokine ELISA kits (Elabscience, China).

### Parasite infection challenge study

Fifty-four female BALB/c mice were randomly divided into six groups (n = 9). Four groups were immunized via intramuscular injection in the thigh with vaccines (20 μg), including IL-4-EG95 mRNA-LNP group, EG95-EC95 mRNA-LNP group, EG95 mRNA-LNP group and CV. Two groups were immunized as the same procedure with LNP (20 μg) and PBS (100 μL) as control. Protection efficacy was evaluated 28 days after the booster immunization of the vaccines through challenge with *E. granulosus* protoscoleces infection. Briefly, hydatid cysts were aseptically collected from the livers of naturally infected sheep at a slaughterhouse in Qinghai Province, China, and transported to the laboratory under cold-chain conditions. Cyst fluid containing protoscoleces was aspirated using a 20 mL syringe, allowed to sediment, and repeatedly washed with PBS (pH 7.4) containing 100 µg/mL streptomycin and penicillin (Solarbio, China) until the supernatant was clear. DNA of protoscoleces was extracted using QIAmp DNA FFPE Tissue Kit (Qiagen, US). Molecular identification was undertaken by PCR-amplifying the complete fragments of the mitochondrial NADH dehydrogenase subunit 1 (*nad1*) gene. The primers were designed based on the multiple sequence alignment of *E. granulosus* complete mt genomes deposited in the National Center for Biotechnology Information (NCBI) GenBank: NC_012896. (*nad1*-F: 5’-ATT ATA GAA AAT TTT CGT TTT ACA CGA-3’, *nad1*-R: 5’-ATT CAC AAT TTA CTA TAT CAA AGT AAC C-3’). After microscopic examination and counting, the mice in six groups was intraperitoneally inoculated with 1000 viable protoscoleces, including IL-4-EG95 group, EG95-EC95 group, EG95 group, CV group, LNP group, and PBS (negative control) group. Cysts development in the mice was assessed six months post-protoscoleces inoculation. The inhibition rate of cysts wet weight and cysts burden were calculated using the formulae, the inhibition rate of wet weight = (Mean cysts wet weight of control group - cysts wet weight of experimental group) / Mean cysts wet weight of control group×100%, the hydatid cysts reduction rate = (Mean cysts count of control group - cysts count of experimental group) / Mean cysts count of control group×100%. The mice liver index and spleen index were calculated using the formulae, liver index = the liver weight (mg) / the body weight (g), spleen index = the spleen weight (mg) / the body weight (g).

### Evaluation of in vitro protoscolicidal efficacy

Protoscoleces of *Echinococcus granulosus* were obtained and processed as described above. The protoscoleces were cultured in DMEM medium supplemented with 100 µg/mL streptomycin and penicillin (Solarbio, China). Following microscopic examination and counting, 300 protoscoleces per well were seeded into 96-well plates. Sera collected from sheep 14 days after the second immunization were used to assess protoscolicidal activity. The following treatment groups were included: IL-4-EG95, EG95-EC95, EG95, CV, PBS (negative control), and NC (untreated control). Sera were tested at concentrations of 10%, 25%, and 50%. Co-incubation was carried out at 37 °C with 5% CO_2_. At 6, 12, 24, and 48 h post-treatment, viability was assessed by fluorescence staining. Fluorescein diacetate (FDA, MCE, USA) was used to stain viable protoscoleces, while propidium iodide (PI, MCE, USA) was used to stain non‑viable (killed) protoscoleces, and the mortality rate was calculated accordingly.

### Statistical analysis

Data were analyzed using GraphPad Prism 8 (GraphPad Software, USA), ImageJ (Germany) and Origin 2021 (OriginLab, USA). All numerical values are expressed as mean ± standard deviation (S.D.). Statistical significance was determined by one-way ANOVA followed by Tukey’s post hoc test. A value of *P* < 0.05 was considered statistically significant.

## Conclusion

In this study, we successfully developed three EG95-based mRNA-LNP vaccines, EG95 mRNA-LNP, EG95-EC95 mRNA-LNP and IL-4-EG95 mRNA-LNP vaccine. A comprehensive evaluation revealed that these mRNA vaccines elicited markedly enhanced cellular and humoral immune responses in animals, along with superior protective efficacy, compared to a commercially available EG95 subunit vaccine. Biodistribution studies displayed excellent biocompatibility of the LNP-based mRNA delivery system. And no adverse effects of the mRNA-LNP vaccines were observed in safety assessments in mice. T/B cell proliferation assays indicated that these mRNA-LNP vaccines induced robust cellular immunity, leading to the generation of potent effector and memory cells. Longitudinal monitoring showed that specific protective antibodies remained at high levels for over 12 months in mRNA-LNP vaccines immunized mice. Parasite infection challenge experiments further demonstrated that these three mRNA-LNP vaccines conferred high levels of protection, significantly inhibited the survival of protoscoleces and the development of cysts. These results suggest that the mRNA vaccine platform outperforms conventional protein-based vaccines and represent a promising candidate for future practical application. In summary, we designed, produced, and comprehensively evaluated three mRNA vaccines targeting *E. granulosus* metacestodes, highlighting their potential as an effective strategy to mitigate the zoonotic threat posed by this parasite.

## Supporting information

S1 Raw Images Original, uncropped images for all blots and gels. This file contains the original, uncropped gel and imprint images, corresponding to Fig 2B, Fig 2C and Fig 2D in the manuscript. Each image is annotated with sample identity, lane order, the corresponding manuscript figure panel, and the positions of molecular weight markers.
(PDF)

S2 Table Physicochemical characterization of mRNA-loaded and empty lipid nanoparticles (LNPs). Size and PDI were measured by dynamic light scattering (DLS). Z-average is the intensity-weighted harmonic mean hydrodynamic diameter determined by cumulants analysis of the correlation function. Polydispersity index (PDI) is a dimensionless measure of the width of the size distribution, calculated from the second cumulant of the correlation function. Data are presented as mean ± SD (n = 3).
(DOCX)

S3 Fig Serum cytokine levels of IFN-γ, IL-2, IL-4 and IL-10 in different vaccines immunized mice. (TIF)

S4 Fig Viability assessment and genotype identification of *E. granulosus* protoscoleces. (A) Microscopic image of protoscoleces isolated from hydatid cysts. (B) Viability assay of protoscoleces determined by Trypan blue exclusion test. (C) PCR amplified *nad*1 gene (1,208 bp) of *E. granulosus* examined in 1% (w/v) agarose gels, (Lanes 1–2: protoscoleces isolated from different hydatid cysts).
(TIF)

S5 Fig Original images of cysts, livers and spleesin different groups of mice, including IL-4-EG95, EG95-EC95, EG95, CV, PBS and NC group. Mice were infected with protoscoleces 28 days after booster immunization of different vaccines and euthanized six months later. The livers, spleens and all the visible cysts of the mice were collected.
(TIF)

S6 Fig In vitro protoscolicidal activity of immune sheep serum. FDA/PI fluorescent staining images of protoscoleces after being treated with sheep serum of IL-4 EG95, EG95-EC95, EG95, CV, PBS, and NC groups for 6 h. Three sheep serum concentrations in the protoscoleces cultured medium were employed, included 10%, 25%, and 50%. Scale bar = 500 μm.
(TIF)

S7 Fig In vitro protoscolicidal activity of immune sheep serum. FDA/PI fluorescent staining images of protoscoleces after being treated with sheep serum of IL-4 EG95, EG95-EC95, EG95, CV, PBS, and NC groups for 12 h. Three sheep serum concentrations in the protoscoleces cultured medium were employed, included 10%, 25%, and 50%. Scale bar = 500 μm.
(TIF)

S8 Fig In vitro protoscolicidal activity of immune sheep serum. FDA/PI fluorescent staining images of protoscoleces after being treated with sheep serum of IL-4 EG95, EG95-EC95, EG95, CV, PBS, and NC groups for 24 h. Three sheep serum concentrations in the protoscoleces cultured medium were employed, included 10%, 25%, and 50%. Scale bar = 500 μm.
(TIF)

S9 Fig In vitro protoscolicidal activity of immune sheep serum. FDA/PI fluorescent staining images of protoscoleces after being treated with sheep serum of IL-4 EG95, EG95-EC95, EG95, CV, PBS, and NC groups for 48 h. Three sheep serum concentrations in the protoscoleces cultured medium were employed, included 10%, 25%, and 50%. Scale bar = 500 μm.
(TIF)

S10 Fig Mortality rate of the protoscoleces after being treated with sheep serum of IL-4 EG95, EG95-EC95, EG95, CV, PBS, and NC groups for different time. Three sheep serum concentrations in the protoscoleces cultured medium were employed, included 10%, 25%, and 50%. Data are presented as means ± SD (n = 3). **** *P* < 0.0001.
(TIF)

S11 Fig Anti-EG95 IgG antibody levers in mice after booster immunization. (TIF)

S12 Fig Rectal temperature monitoring in sheep post-immunization. Data are presented as means ± SD (n = 7).
(TIF)

## References

[1] Larrieu E, Mujica G, Araya D, Labanchi JL, Arezo M, Herrero E, et al. Pilot field trial of the EG95 vaccine against ovine cystic echinococcosis in Rio Negro, Argentina: 8 years of work. Acta Trop. 2019;191:1–7. doi: 10.1016/j.actatropica.2018.12.025 30576624

[2] Li J, Wei J. Trends in the disease burden of cystic echinococcosis in China, 1990-2044 analysis and forecasting study. Sci Rep. 2025;15(1):4812. doi: 10.1038/s41598-025-88403-8 39924567 PMC11808116

[3] Agudelo Higuita NI, Brunetti E, McCloskey C. Cystic Echinococcosis. J Clin Microbiol. 2016;54(3): 518–23. 26677245 10.1128/JCM.02420-15 PMC4767951

[4] da Silva AM. Human echinococcosis: A neglected disease. Gastroenterol Res Pract. 2010;2010:583297. doi: 10.1155/2010/583297 20862339 PMC2939407

[5] Manterola C, Rojas C, Totomoch-Serra A, García-Méndez N, Riffo-Campos ÁL. *Echinococcus granulosus* genotypes verified in human hydatid disease around the world. Systematic review. Rev Chilena Infectol. 2020;37(5):541–9. doi: 10.4067/S0716-10182020000500541 33399801

[6] Tamarozzi F, Deplazes P, Casulli A. Reinventing the wheel of *Echinococcus granulosus* sensu lato transmission to humans. Trends Parasitol. 2020;36(5):427–34. doi: 10.1016/j.pt.2020.02.004 32298630

[7] Gauci C, Heath D, Chow C, Lightowlers MW. Hydatid disease: vaccinology and development of the EG95 recombinant vaccine. Expert Rev Vaccines. 2005;4(1):103–12. doi: 10.1586/14760584.4.1.103 15757477

[8] Chow C, Gauci CG, Cowman AF, Lightowlers MW. *Echinococcus granulosus*: oncosphere-specific transcription of genes encoding a host-protective antigen. Exp Parasitol. 2004;106(3–4):183–6. doi: 10.1016/j.exppara.2004.03.009 15172227

[9] Lightowlers MW, Heath DD. Immunity and vaccine control of *Echinococcus granulosus* infection in animal intermediate hosts. Parassitologia. 2004;46(1–2):27–31. 15305682

[10] Jiang T, Mahemuti M, Wang W, Han S, Wu X, Liu H, et al. Development and protective efficacy of multi-epitope vaccine FL46 against cystic echinococcosis. Front Immunol. 2025;16:1686959. doi: 10.3389/fimmu.2025.1686959 41181113 PMC12571753

[11] Shao G, Zhu X, Hua R, Lu Z, Chen Y, Yang A, et al. Cocktail vaccine induces immunoprotection and modulates the fecal microbiota in dogs against *Echinococcus granulosus* infection. NPJ Vaccines. 2025;10(1):214. doi: 10.1038/s41541-025-01275-x 41034249 PMC12488870

[12] Korhonen PK, Kinkar L, Young ND, Cai H, Lightowlers MW, Gauci C, et al. Chromosome-scale *Echinococcus granulosus* (genotype G1) genome reveals the Eg95 gene family and conservation of the EG95-vaccine molecule. Commun Biol. 2022;5(1):199. doi: 10.1038/s42003-022-03125-1 35241789 PMC8894454

[13] Jabbar A, Jenkins DJ, Crawford S, Walduck AK, Gauci CG, Lightowlers MW. Oncospheral penetration glands are the source of the EG95 vaccine antigen against cystic hydatid disease. Parasitology. 2011;138(1):89–99. doi: 10.1017/S0031182010001034 20663253

[14] Gauci C, Merli M, Muller V, Chow C, Yagi K, Mackenstedt U, et al. Molecular cloning of a vaccine antigen against infection with the larval stage of *Echinococcus multilocularis*. Infect Immun. 2002;70(7):3969–72. doi: 10.1128/IAI.70.7.3969-3972.2002 12065546 PMC128094

[15] Lightowlers MW, Lawrence SB, Gauci CG, Young J, Ralston MJ, Maas D, et al. Vaccination against hydatidosis using a defined recombinant antigen. Parasite Immunol. 1996;18(9):457–62. doi: 10.1111/j.1365-3024.1996.tb01029.x 9226681

[16] Huang F, Dang Z, Suzuki Y, Horiuchi T, Yagi K, Kouguchi H, et al. Analysis on gene expression profile in oncospheres and early stage metacestodes from *Echinococcus multilocularis*. PLoS Negl Trop Dis. 2016;10(4):e0004634. doi: 10.1371/journal.pntd.0004634 27092774 PMC4836691

[17] Tan JL, Ueda N, Heath D, Mercer AA, Fleming SB. Development of orf virus as a bifunctional recombinant vaccine: surface display of *Echinococcus granulosus* antigen EG95 by fusion to membrane structural proteins. Vaccine. 2012;30(2):398–406. doi: 10.1016/j.vaccine.2011.10.079 22085551

[18] Xu T, Liu L, Shi C, Liu W, Wang M, Tian L, et al. A recombinant rabies virus expressing *Echinococcus granulosus* EG95 induces protective immunity in mice. Transbound Emerg Dis. 2022;69(4):e254–66. doi: 10.1111/tbed.14292 34403194

[19] Liu F, Li L, Liu Y, Sun C, Liu C, Wu X, et al. Development of reverse genetics system for small ruminant morbillivirus: Rescuing recombinant virus to express *Echinococcus granulosus* EG95 antigen. Virus Res. 2019;261:50–5. doi: 10.1016/j.virusres.2018.12.008 30557577

[20] Gauci CG, Jenkins DJ, Lightowlers MW. Protection against cystic echinococcosis in sheep using an *Escherichia coli*-expressed recombinant antigen (EG95) as a bacterin. Parasitology. 2023;150(1):29–31. doi: 10.1017/S0031182022001457 36226566 PMC10090584

[21] Li W, Ou X, He A. Construction, identification, and expression of lactococcus lactis-based recombinant vaccine for *Echinococcus granulosus*. Sichuan Da Xue Xue Bao Yi Xue Ban. 2023;54(6):1154–8. doi: 10.12182/20231160105 38162084 PMC10752785

[22] Gao X, Zhu X, Liu X, Zhou C, Shang Y, Wu T, et al. A ferritin-based Eg95 nanoparticle vaccine adjuvanted with pCpG eliciting robust immune responses against cystic echinococcosis in mice model. Int J Nanomedicine. 2025;20:309–25. doi: 10.2147/IJN.S499938 39802377 PMC11725278

[23] Pourseif MM, Moghaddam G, Naghili B, Saeedi N, Parvizpour S, Nematollahi A, et al. A novel in silico minigene vaccine based on CD4+ T-helper and B-cell epitopes of EG95 isolates for vaccination against cystic echinococcosis. Comput Biol Chem. 2018;72:150–63. doi: 10.1016/j.compbiolchem.2017.11.008 29195784

[24] Zhao Y, Bi Q, Wei Y, Wang R, Wang G, Fu G, et al. A DNA vaccine (EG95-PT1/2/3-IL2) encoding multi-epitope antigen and IL-2 provokes efficient and long-term immunity to echinococcosis. J Control Release. 2023;361:402–16. 37527761 10.1016/j.jconrel.2023.07.047

[25] Xie C, Yao R, Xia X. The advances of adjuvants in mRNA vaccines. NPJ Vaccines. 2023;8(1):162. doi: 10.1038/s41541-023-00760-5 37884526 PMC10603121

[26] Sahin U, Karikó K, Türeci Ö. mRNA-based therapeutics--developing a new class of drugs. Nat Rev Drug Discov. 2014;13(10):759–80. doi: 10.1038/nrd4278 25233993

[27] Le T, Sun C, Chang J, Zhang G, Yin X. mRNA vaccine development for emerging animal and zoonotic diseases. Viruses. 2022;14(2):401. doi: 10.3390/v14020401 35215994 PMC8877136

[28] Anand P, Stahel VP. Correction to: The safety of Covid-19 mRNA vaccines: A review. Patient safety in surgery. 2021;15(1):22. 34006292 10.1186/s13037-021-00296-4 PMC8130782

[29] Pardi N, Parkhouse K, Kirkpatrick E, McMahon M, Zost SJ, Mui BL, et al. Nucleoside-modified mRNA immunization elicits influenza virus hemagglutinin stalk-specific antibodies. Nat Commun. 2018;9(1):3361. doi: 10.1038/s41467-018-05482-0 30135514 PMC6105651

[30] Richner JM, Himansu S, Dowd KA, Butler SL, Salazar V, Fox JM, et al. Modified mRNA vaccines protect against Zika virus infection. Cell. 2017;168(6):1114-1125.e10. doi: 10.1016/j.cell.2017.02.017 28222903 PMC5388441

[31] Lutz J, Lazzaro S, Habbeddine M, Schmidt KE, Baumhof P, Mui BL, et al. Unmodified mRNA in LNPs constitutes a competitive technology for prophylactic vaccines. NPJ Vaccines. 2017;2:29. doi: 10.1038/s41541-017-0032-6 29263884 PMC5648897

[32] Raj DK, Das Mohapatra A, Jnawali A, Zuromski J, Jha A, Cham-Kpu G, et al. Anti-PfGARP activates programmed cell death of parasites and reduces severe malaria. Nature. 2020;582(7810):104–8. doi: 10.1038/s41586-020-2220-1 32427965 PMC7372601

[33] Wu Q, Zhang Z, Chu H, Xia B, Li W, Ding J, et al. A novel combined quadrivalent self-amplifying mRNA-LNP vaccine provokes protective immunity against acute and chronic toxoplasmosis in mice. Infect Dis Poverty. 2025;14(1):55. doi: 10.1186/s40249-025-01332-6 40551280 PMC12183821

[34] Zhang Y, Li D, Shen Y, Li S, Lu S, Zheng B. Immunization with a novel mRNA vaccine, TGGT1_ 216 200 mRNA-LNP, prolongs survival time in BALB/c mice against acute toxoplasmosis. Frontiers in Immunol. 2023;14:1161507. 37122740 10.3389/fimmu.2023.1161507 PMC10140528

[35] Duthie MS, Van Hoeven N, MacMillen Z, Picone A, Mohamath R, Erasmus J, et al. Heterologous immunization with defined RNA and subunit vaccines enhances T cell responses that protect against *Leishmania donovani*. Front Immunol. 2018;9:2420. doi: 10.3389/fimmu.2018.02420 30386348 PMC6199377

[36] Pardi N, Hogan MJ, Porter FW, Weissman D. mRNA vaccines - a new era in vaccinology. Nat Rev Drug Discov. 2018;17(4):261–79. doi: 10.1038/nrd.2017.243 29326426 PMC5906799

[37] Teijaro JR, Farber DL. COVID-19 vaccines: modes of immune activation and future challenges. Nat Rev Immunol. 2021;21(4):195–7. doi: 10.1038/s41577-021-00526-x 33674759 PMC7934118

[38] Junttila IS. Tuning the cytokine responses: An update on Interleukin (IL)-4 and IL-13 receptor complexes. Front Immunol. 2018;9:888. doi: 10.3389/fimmu.2018.00888 29930549 PMC6001902

[39] Deplazes P, Rinaldi L, Alvarez Rojas CA, Torgerson PR, Harandi MF, Romig T, et al. Global distribution of alveolar and cystic echinococcosis. Adv Parasitol. 2017;95:315–493. doi: 10.1016/bs.apar.2016.11.001 28131365

[40] Casulli A, Massolo A, Saarma U, Umhang G, Santolamazza F, Santoro A. Species and genotypes belonging to *Echinococcus granulosus* sensu lato complex causing human cystic echinococcosis in Europe (2000-2021): A systematic review. Parasit Vectors. 2022;15(1):109. doi: 10.1186/s13071-022-05197-8 35346335 PMC8962544

[41] Anand N, Sehgal R, Kanwar RK, Dubey ML, Vasishta RK, Kanwar JR. Oral administration of encapsulated bovine lactoferrin protein nanocapsules against intracellular parasite *Toxoplasma gondii*. Int J Nanomedicine. 2015;10:6355–69. doi: 10.2147/IJN.S85286 26504384 PMC4605239

[42] Nemati S, Mottaghi M, Karami P, Mirjalali H. Development of solid lipid nanoparticles-loaded drugs in parasitic diseases. Discov Nano. 2024;19(1):7. doi: 10.1186/s11671-023-03955-w 38175309 PMC10767167

[43] Bevers S, Kooijmans SAA, Van de Velde E, Evers MJW, Seghers S, Gitz-Francois JJJM, et al. mRNA-LNP vaccines tuned for systemic immunization induce strong antitumor immunity by engaging splenic immune cells. Mol Ther. 2022;30(9):3078–94. doi: 10.1016/j.ymthe.2022.07.007 35821637 PMC9273295

[44] Soroudi S, Jaafari MR, Arabi L. Lipid nanoparticle (LNP) mediated mRNA delivery in cardiovascular diseases: Advances in genome editing and CAR T cell therapy. J Control Release. 2024;372:113–40. doi: 10.1016/j.jconrel.2024.06.023 38876358

[45] Huang X, Ma Y, Ma G, Xia Y. Unlocking the therapeutic applicability of LNP-mRNA: Chemistry, formulation, and clinical strategies. Research (Wash D C). 2024;7:0370. doi: 10.34133/research.0370 38894715 PMC11185168

[46] Kdimati S, Mullins CS, Linnebacher M. Cancer-cell-derived IgG and its potential role in tumor development. Int J Mol Sci. 2021;22(21):11597. doi: 10.3390/ijms222111597 34769026 PMC8583861

[47] Hussell T, Spender LC, Georgiou A, O’Garra A, Openshaw PJ. Th1 and Th2 cytokine induction in pulmonary T cells during infection with respiratory syncytial virus. J Gen Virol. 1996;77 (Pt 10):2447–55. doi: 10.1099/0022-1317-77-10-2447 8887477

[48] Karaji N, Sattentau QJ. Efferocytosis of pathogen-infected cells. Front Immunol. 2017;8:1863. doi: 10.3389/fimmu.2017.01863 29312342 PMC5743670

[49] Heinzel S, Marchingo JM, Horton MB, Hodgkin PD. The regulation of lymphocyte activation and proliferation. Curr Opin Immunol. 2018;51:32–8. doi: 10.1016/j.coi.2018.01.002 29414529

[50] Lin Y-J, Goretzki A, Schülke S. Immune metabolism of IL-4-activated B Cells and Th2 Cells in the context of allergic diseases. Front Immunol. 2021;12:790658. doi: 10.3389/fimmu.2021.790658 34925372 PMC8671807

[51] Valzasina B, Piconese S, Guiducci C, Colombo MP. Tumor-induced expansion of regulatory T cells by conversion of CD4+CD25- lymphocytes is thymus and proliferation independent. Cancer Res. 2006;66(8):4488–95. doi: 10.1158/0008-5472.CAN-05-4217 16618776

[52] Ruterbusch M, Pruner KB, Shehata L, Pepper M. In vivo CD4+ T cell differentiation and function: Revisiting the Th1/Th2 paradigm. Annu Rev Immunol. 2020;38:705–25. doi: 10.1146/annurev-immunol-103019-085803 32340571

[53] Powell MD, Read KA, Sreekumar BK, Oestreich KJ. Ikaros Zinc finger transcription factors: regulators of cytokine signaling pathways and CD4+ T helper cell differentiation. Front Immunol. 2019;10:1299. doi: 10.3389/fimmu.2019.01299 31244845 PMC6563078

[54] Reina-Campos M, Scharping NE, Goldrath AW. CD8+ T cell metabolism in infection and cancer. Nat Rev Immunol. 2021;21(11):718–38. doi: 10.1038/s41577-021-00537-8 33981085 PMC8806153

[55] Xia J, Fu Y, Huang W, Uddin T, Sibley LD. Constitutive upregulation of transcription factors underlies permissive bradyzoite differentiation in a natural isolate of *Toxoplasma gondii*. mBio. 2024;15(9):e0064124. doi: 10.1128/mbio.00641-24 39150246 PMC11389365

[56] Wu F, Ding X-Y, Li X-H, Gong M-J, An J-Q, Huang S-L. Correlation between elevated inflammatory cytokines of spleen and spleen index in acute spinal cord injury. J Neuroimmunol. 2020;344:577264. doi: 10.1016/j.jneuroim.2020.577264 32447026

[57] Furuichi Y, Fujiwara T, Shimojima R, Sato K, Kato H. Enlargement of the spleen index is a predictor of the occurrence of esophageal varices and hepatocellular carcinoma after administering direct-acting antiviral agents. Intern Med. 2023;62(18):2597–606. doi: 10.2169/internalmedicine.1166-22 36725036 PMC10569918

[58] Stahlschmidt FL, Tafarel JR, Menini-Stahlschmidt CM, Baena CP. Hepatorenal index for grading liver steatosis with concomitant fibrosis. PLoS One. 2021;16(2):e0246837. doi: 10.1371/journal.pone.0246837 33577616 PMC7880490

[59] Huttman M, Parigi TL, Zoncapè M, Liguori A, Kalafateli M, Noel-Storr AH. Cochrane Database System Rev. 2024;8(8):Cd011929. 39136280 10.1002/14651858.CD011929.pub2 PMC11320661

[60] Jackson LA, Anderson EJ, Rouphael NG, Roberts PC, Makhene M, Coler RN, et al. An mRNA vaccine against SARS-CoV-2 - preliminary report. N Engl J Med. 2020;383(20):1920–31. doi: 10.1056/NEJMoa2022483 32663912 PMC7377258

[61] de Alwis R, Gan ES, Chen S, Leong YS, Tan HC, Zhang SL, et al. A single dose of self-transcribing and replicating RNA-based SARS-CoV-2 vaccine produces protective adaptive immunity in mice. Mol Ther. 2021;29(6):1970–83. doi: 10.1016/j.ymthe.2021.04.001 33823303 PMC8019652

[62] Corbett KS, Flynn B, Foulds KE, Francica JR, Boyoglu-Barnum S, Werner AP, et al. Evaluation of the mRNA-1273 vaccine against SARS-CoV-2 in Nonhuman primates. N Engl J Med. 2020;383(16):1544–55. doi: 10.1056/NEJMoa2024671 32722908 PMC7449230

[63] Pardi N, Parkhouse K, Kirkpatrick E, McMahon M, Zost SJ, Mui BL, et al. Nucleoside-modified mRNA immunization elicits influenza virus hemagglutinin stalk-specific antibodies. Nat Commun. 2018;9(1):3361. doi: 10.1038/s41467-018-05482-0 30135514 PMC6105651

[64] Bahl K, Senn JJ, Yuzhakov O, Bulychev A, Brito LA, Hassett KJ, et al. Preclinical and clinical demonstration of immunogenicity by mRNA vaccines against H10N8 and H7N9 influenza viruses. Mol Ther. 2017;25(6):1316–27. doi: 10.1016/j.ymthe.2017.03.035 28457665 PMC5475249

[65] Alberer M, Gnad-Vogt U, Hong HS, Mehr KT, Backert L, Finak G, et al. Safety and immunogenicity of a mRNA rabies vaccine in healthy adults: an open-label, non-randomised, prospective, first-in-human phase 1 clinical trial. Lancet. 2017;390(10101):1511–20. doi: 10.1016/S0140-6736(17)31665-3 28754494

[66] Chahal JS, Khan OF, Cooper CL, McPartlan JS, Tsosie JK, Tilley LD, et al. Dendrimer-RNA nanoparticles generate protective immunity against lethal Ebola, H1N1 influenza, and *Toxoplasma gondii* challenges with a single dose. Proc Natl Acad Sci U S A. 2016;113(29):E4133-42. doi: 10.1073/pnas.1600299113 27382155 PMC4961123

[67] Hodžić A, Alić A, Spahić A, Harl J, Beck R. Genetic diversity of *Echinococcus granulosus* sensu lato from animals and humans in Bosnia and Herzegovina. Parasit Vectors. 2022;15(1):457. doi: 10.1186/s13071-022-05598-9 36482478 PMC9733143

[68] Hua R-Q, Du X-D, He X, Gu X-B, Xie Y, He R, et al. Genetic diversity of Echinococcus granulosus sensu lato in China: Epidemiological studies and systematic review. Transbound Emerg Dis. 2022;69(5):e1382–92. doi: 10.1111/tbed.14469 35139582

[69] Bonelli P, Dei Giudici S, Peruzzu A, Piseddu T, Santucciu C, Masu G, et al. Genetic diversity of *Echinococcus granulosus* sensu stricto in Sardinia (Italy). Parasitol Int. 2020;77:102120. doi: 10.1016/j.parint.2020.102120 32259586

[70] Cucher MA, Macchiaroli N, Baldi G, Camicia F, Prada L, Maldonado L, et al. Cystic echinococcosis in South America: Systematic review of species and genotypes of Echinococcus granulosus sensu lato in humans and natural domestic hosts. Trop Med Int Health. 2016;21(2):166–75. doi: 10.1111/tmi.12647 26610060

[71] McMahon M, O’Dell G, Tan J, Sárközy A, Vadovics M, Carreño JM, et al. Assessment of a quadrivalent nucleoside-modified mRNA vaccine that protects against group 2 influenza viruses. Proc Natl Acad Sci U S A. 2022;119(45):e2206333119. doi: 10.1073/pnas.2206333119 36322769 PMC9659346

[72] Freyn AW, Ramos da Silva J, Rosado VC, Bliss CM, Pine M, Mui BL, et al. A multi-targeting, nucleoside-modified mRNA influenza virus vaccine provides broad protection in mice. Mol Ther. 2020;28(7):1569–84. doi: 10.1016/j.ymthe.2020.04.018 32359470 PMC7335735

[73] Pardi N, Carreño JM, O’Dell G, Tan J, Bajusz C, Muramatsu H, et al. Development of a pentavalent broadly protective nucleoside-modified mRNA vaccine against influenza B viruses. Nat Commun. 2022;13(1):4677. doi: 10.1038/s41467-022-32149-8 35945226 PMC9362976

[74] Kackos CM, DeBeauchamp J, Davitt CJH, Lonzaric J, Sealy RE, Hurwitz JL, et al. Seasonal quadrivalent mRNA vaccine prevents and mitigates influenza infection. NPJ Vaccines. 2023;8(1):157. doi: 10.1038/s41541-023-00752-5 37828126 PMC10570305

[75] Read AJ, Casey JL, Coley AM, Foley M, Gauci CG, Jackson DC, et al. Isolation of antibodies specific to a single conformation-dependant antigenic determinant on the EG95 hydatid vaccine. Vaccine. 2009;27(7):1024–31. doi: 10.1016/j.vaccine.2008.11.096 19095030 PMC2670974

[76] Wang L, Gao J, Lan X, Zhao H, Shang X, Tian F, et al. Identification of combined T-cell and B-cell reactive *Echinococcus granulosus* 95 antigens for the potential development of a multi-epitope vaccine. Ann Transl Med. 2019;7(22):652. doi: 10.21037/atm.2019.10.87 31930053 PMC6944580

[77] Zhu J, Yamane H, Paul WE. Differentiation of effector CD4 T cell populations (*). Annu Rev Immunol. 2010;28:445–89. doi: 10.1146/annurev-immunol-030409-101212 20192806 PMC3502616

[78] Perrigoue JG, Saenz SA, Siracusa MC, Allenspach EJ, Taylor BC, Giacomin PR, et al. MHC class II-dependent basophil-CD4+ T cell interactions promote T(H)2 cytokine-dependent immunity. Nat Immunol. 2009;10(7):697–705. doi: 10.1038/ni.1740 19465906 PMC2711559

[79] Mountford AP, Hogg KG, Coulson PS, Brombacher F. Signaling via interleukin-4 receptor alpha chain is required for successful vaccination against schistosomiasis in BALB/c mice. Infect Immun. 2001;69(1):228–36. doi: 10.1128/IAI.69.1.228-236.2001 11119510 PMC97876

[80] Lokras AG, Bobak TR, Baghel SS, Sebastiani F, Foged C. Advances in the design and delivery of RNA vaccines for infectious diseases. Adv Drug Deliv Rev. 2024;213:115419. doi: 10.1016/j.addr.2024.115419 39111358

[81] Chandra S, Wilson JC, Good D, Wei MQ. mRNA vaccines: a new era in vaccine development. Oncology Res. 2024;32(10):1543–64. 39308511 10.32604/or.2024.043987 PMC11413818

[82] Alvarez Rojas CA, Gauci CG, Nolan MJ, Harandi MF, Lightowlers MW. Characterization of the eg95 gene family in the G6 genotype of *Echinococcus granulosus*. Mol Biochem Parasitol. 2012;183(2):115–21. doi: 10.1016/j.molbiopara.2012.02.005 22349630

[83] Laurimäe T, Kinkar L, Romig T, Omer RA, Casulli A, Umhang G, et al. The benefits of analysing complete mitochondrial genomes: Deep insights into the phylogeny and population structure of *Echinococcus granulosus* sensu lato genotypes G6 and G7. Infect Genet Evol. 2018;64:85–94. doi: 10.1016/j.meegid.2018.06.016 29906638

[84] Kantor AB, Herzenberg LA. Origin of murine B cell lineages. Annu Rev Immunol. 1993;11:501–38. doi: 10.1146/annurev.iy.11.040193.002441 8476571

[85] Ochsenbein AF, Fehr T, Lutz C, Suter M, Brombacher F, Hengartner H, et al. Control of early viral and bacterial distribution and disease by natural antibodies. Science. 1999;286(5447):2156–9. doi: 10.1126/science.286.5447.2156 10591647

[86] Tao TW, Uhr JW. Primary-type antibody response in vitro. Science. 1966;151(3714):1096–8. doi: 10.1126/science.151.3714.1096 5909782

[87] Boes M, Prodeus AP, Schmidt T, Carroll MC, Chen J. A critical role of natural immunoglobulin M in immediate defense against systemic bacterial infection. J Exp Med. 1998;188(12):2381–6. doi: 10.1084/jem.188.12.2381 9858525 PMC2212438

[88] Baz A, Richieri A, Puglia A, Nieto A, Dematteis S. Antibody response in CD4-depleted mice after immunization or during early infection with *Echinococcus granulosus*. Parasite Immunol. 1999;21(3):141–50. doi: 10.1046/j.1365-3024.1999.00212.x 10205794

[89] Severi MA, Ferragut G, Nieto A. Antibody response of *Echinococcus granulosus* infected mice: Protoscolex specific response during infection is associated with decreasing specific IgG1/IgG3 ratio as well as decreasing avidity. Parasite Immunol. 1997;19(12):545–52. doi: 10.1046/j.1365-3024.1997.d01-172.x 9458466

[90] Culbreth MJ, Biryukov SS, Shoe JL, Dankmeyer JL, Hunter M, Klimko CP, et al. The use of analgesics during vaccination with a live attenuated Yersinia pestis vaccine alters the resulting immune response in mice. Vaccines (Basel). 2019;7(4):205. doi: 10.3390/vaccines7040205 31816945 PMC6963655

[91] Yang K, Whalen BJ, Tirabassi RS, Selin LK, Levchenko TS, Torchilin VP, et al. A DNA vaccine prime followed by a liposome-encapsulated protein boost confers enhanced mucosal immune responses and protection. J Immunol. 2008;180(9):6159–67. doi: 10.4049/jimmunol.180.9.6159 18424737 PMC3633597

[92] Wong CK, Ho CY, Ko FW, Chan CH, Ho AS, Hui DS. Proinflammatory cytokines (IL-17, IL-6, IL-18 and IL-12) and Th cytokines (IFN-gamma, IL-4, IL-10 and IL-13) in patients with allergic asthma. Clin Exp Immunol. 2001;125(2):177–83. 11529906 10.1046/j.1365-2249.2001.01602.x PMC1906135

[93] Cortés A, Muñoz-Antoli C, Esteban JG, Toledo R. Th2 and Th1 responses: Clear and hidden sides of immunity against intestinal helminths. Trends Parasitol. 2017;33(9):678–93. doi: 10.1016/j.pt.2017.05.004 28566191

[94] Shoeran G, Anand N. Interplay of autophagy and Th1/Th2-mediated macrophage polarization in host-pathogen dynamics. Front Cell Infect Microbiol. 2025;15:1679514. doi: 10.3389/fcimb.2025.1679514 41112570 PMC12528116

[95] Chen TT, Tao MH, Levy R. Idiotype-cytokine fusion proteins as cancer vaccines. Relative efficacy of IL-2, IL-4, and granulocyte-macrophage colony-stimulating factor. J Immunol. 1994;153(10):4775–87. doi: 10.4049/jimmunol.153.10.4775 7525715

[96] Rostami-Rad S, Jafari R, Yousofi Darani H. Th1/Th2-type cytokine profile in C57 black mice inoculated with live *Echinococcus granulosus* protoscolices. J Infect Public Health. 2018;11(6):834–9. doi: 10.1016/j.jiph.2018.06.007 30033229

[97] Tan F, Zhang L, Yin L, Wang L, Zhang H, Zheng L, et al. Immune synergistic mechanism of recombinant plasmid adjuvant containing chicken IL-4 and IL-2 fusion genes on chicken coccidia live vaccine. Poult Sci. 2024;103(1):103204. doi: 10.1016/j.psj.2023.103204 37939587 PMC10665987

[98] Koch T, Mellinghoff SC, Shamsrizi P, Addo MM, Dahlke C. Correlates of vaccine-induced protection against SARS-CoV-2. Vaccines (Basel). 2021;9(3):238. doi: 10.3390/vaccines9030238 33801831 PMC8035658

[99] McAdams D, Estrada M, Holland D, Singh J, Sawant N, Hickey JM, et al. Concordance of in vitro and in vivo measures of non-replicating rotavirus vaccine potency. Vaccine. 2022;40(34):5069–78. doi: 10.1016/j.vaccine.2022.07.017 35871866 PMC9405915

[100] Mittrücker H-W, Visekruna A, Huber M. Heterogeneity in the differentiation and function of CD8⁺ T cells. Arch Immunol Ther Exp (Warsz). 2014;62(6):449–58. doi: 10.1007/s00005-014-0293-y 24879097

[101] Santos JHM, Siddle HV, Raza A, Stanisic DI, Good MF, Tabor AE. Exploring the landscape of Babesia bovis vaccines: Progress, challenges, and opportunities. Parasit Vectors. 2023;16(1):274. doi: 10.1186/s13071-023-05885-z 37563668 PMC10413621

[102] Antonio-Nkondjio C, Sandjo NN, Awono-Ambene P, Wondji CS. Implementing a larviciding efficacy or effectiveness control intervention against malaria vectors: Key parameters for success. Parasit Vectors. 2018;11(1):57. doi: 10.1186/s13071-018-2627-9 29368633 PMC5784718

[103] Woollard DJ, Gauci CG, Heath DD, Lightowlers MW. Protection against hydatid disease induced with the EG95 vaccine is associated with conformational epitopes. Vaccine. 2000;19(4–5):498–507. doi: 10.1016/s0264-410x(00)00192-4 11027814

[104] Woollard DJ, Gauci CG, Lightowlers MW. Synthetic peptides induce antibody against a host-protective antigen of *Echinococcus granulosus*. Vaccine. 1999;18(9–10):785–94. doi: 10.1016/s0264-410x(99)00350-3 10580190

[105] Su L-J, Wang N-N, Luo R, Ji Z-H, Gu H, Yang C, et al. Artificial intelligence-guided design of LNPs for in vivo targeted mRNA delivery via analysis of the spatial conformation of ionizable lipids. Nat Biomed Eng. 2026;10.1038/s41551-026-01640–8. doi: 10.1038/s41551-026-01640-8 41851281

[106] Kis Z, Tak K, Ibrahim D, Papathanasiou MM, Chachuat B, Shah N, et al. Pandemic-response adenoviral vector and RNA vaccine manufacturing. NPJ Vaccines. 2022;7(1):29. doi: 10.1038/s41541-022-00447-3 35236838 PMC8891260

[107] Yu P-C, Dan M, He Y, Meng S-L, Yang H-Y, Su X, et al. A novel mRNA rabies vaccine as a promising candidate for rabies post-exposure prophylaxis protects animals from different rabies viruses. Microb Pathog. 2023;185:106425. doi: 10.1016/j.micpath.2023.106425 37923181

[108] Pourseif MM, Moghaddam G, Saeedi N, Barzegari A, Dehghani J, Omidi Y. Current status and future prospective of vaccine development against *Echinococcus granulosus*. Biologicals. 2018;51:1–11. doi: 10.1016/j.biologicals.2017.10.003 29100669

[109] Laskowski RA, Thornton JM. PDBsum extras: SARS-CoV-2 and AlphaFold models. Protein Sci. 2022;31(1):283–9. doi: 10.1002/pro.4238 34779073 PMC8662102

[110] Kitidee K, Amonyingcharoen S, Preedagasamzin S, Atjanasuppat K, Sawaisorn P, Srimorkun P, et al. Combining CD3/GD2 bispecific T cell engager with human Vγ9Vδ2 T cells facilitates neuroblastoma cell targeting and killing in vitro. PLoS One. 2025;20(6):e0325389. doi: 10.1371/journal.pone.0325389 40489497 PMC12148185

[111] Kim J, Jozic A, Mukherjee A, Nelson D, Chiem K, Khan MSR, et al. Rapid generation of circulating and mucosal decoy human ACE2 using mRNA nanotherapeutics for the potential treatment of SARS-CoV-2. Adv Sci (Weinh). 2022;9(35):e2202556. doi: 10.1002/advs.202202556 36216580 PMC9762296

[112] Yang S-T, Kreutzberger AJB, Lee J, Kiessling V, Tamm LK. The role of cholesterol in membrane fusion. Chem Phys Lipids. 2016;199:136–43. doi: 10.1016/j.chemphyslip.2016.05.003 27179407 PMC4972649

[113] Paunovska K, Da Silva Sanchez AJ, Sago CD, Gan Z, Lokugamage MP, Islam FZ, et al. Nanoparticles containing oxidized cholesterol deliver mRNA to the liver microenvironment at clinically relevant doses. Adv Mater. 2019;31(14):e1807748. doi: 10.1002/adma.201807748 30748040 PMC6445717

[114] Lee HE, Yang G, Choi JS, Lee JY. Suppression of primary splenocyte proliferation by artemisia capillaris and its components. Toxicol Res. 2017;33(4):283–90. doi: 10.5487/TR.2017.33.4.283 29071012 PMC5654196

[115] Basta-Kaim A, Budziszewska B, Jagła G, Nowak W, Kubera M, Lasoń W. Inhibitory effect of antipsychotic drugs on the Con A- and LPS-induced proliferative activity of mouse splenocytes: A possible mechanism of action. J Physiol Pharmacol. 2006;57(2):247–64. 16845229

[116] Liao W, Spolski R, Li P, Du N, West EE, Ren M, et al. Opposing actions of IL-2 and IL-21 on Th9 differentiation correlate with their differential regulation of BCL6 expression. Proc Natl Acad Sci U S A. 2014;111(9):3508–13. doi: 10.1073/pnas.1301138111 24550509 PMC3948278

