# Supplementary material for: Novel EG95-based mRNA vaccines induce long-term protective immunity against echinococcosis
Source: PLoS Pathog. 2026 Oct 9;22(10):e1014659. doi: 10.1371/journal.ppat.1014659 (PMC13674861; doi:10.1371/journal.ppat.1014659)
Supplement: Supplementary file 1 [file ppat.1014659.s001.pdf]

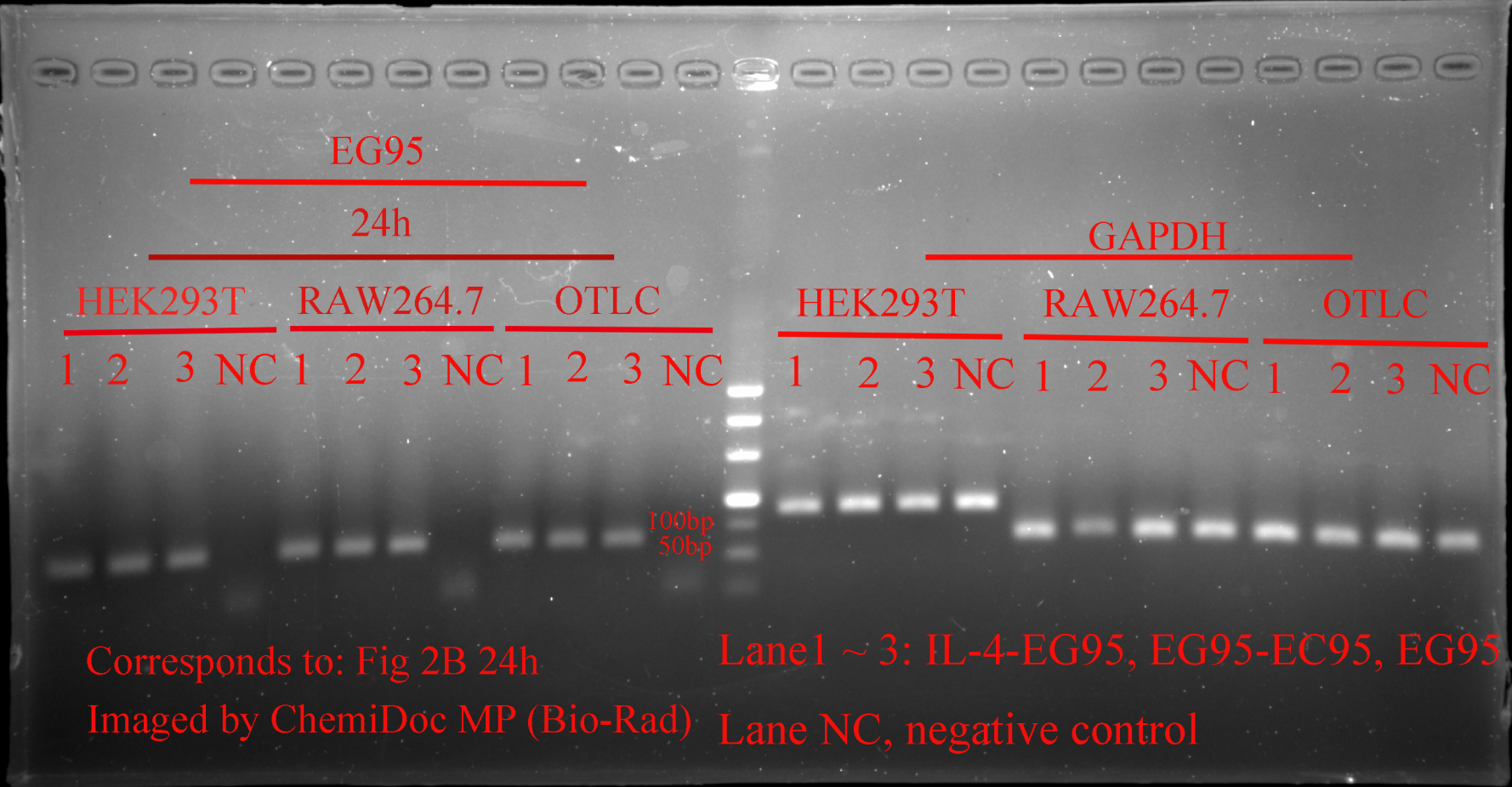
EG95
24h
HEK293T
RAW264.7
OTLC
1 2 3 NC 1 2 3 NC 1 2 3 NC
100bp
50bp
GAPDH
HEK293T
RAW264.7
OTLC
1 2 3 NC 1 2 3 NC 1 2 3 NC
Corresponds to: Fig 2B 24h
Imaged by ChemiDoc MP (Bio-Rad)
Lane1 ~ 3: IL-4-EG95, EG95-EC95, EG95
Lane NC, negative control

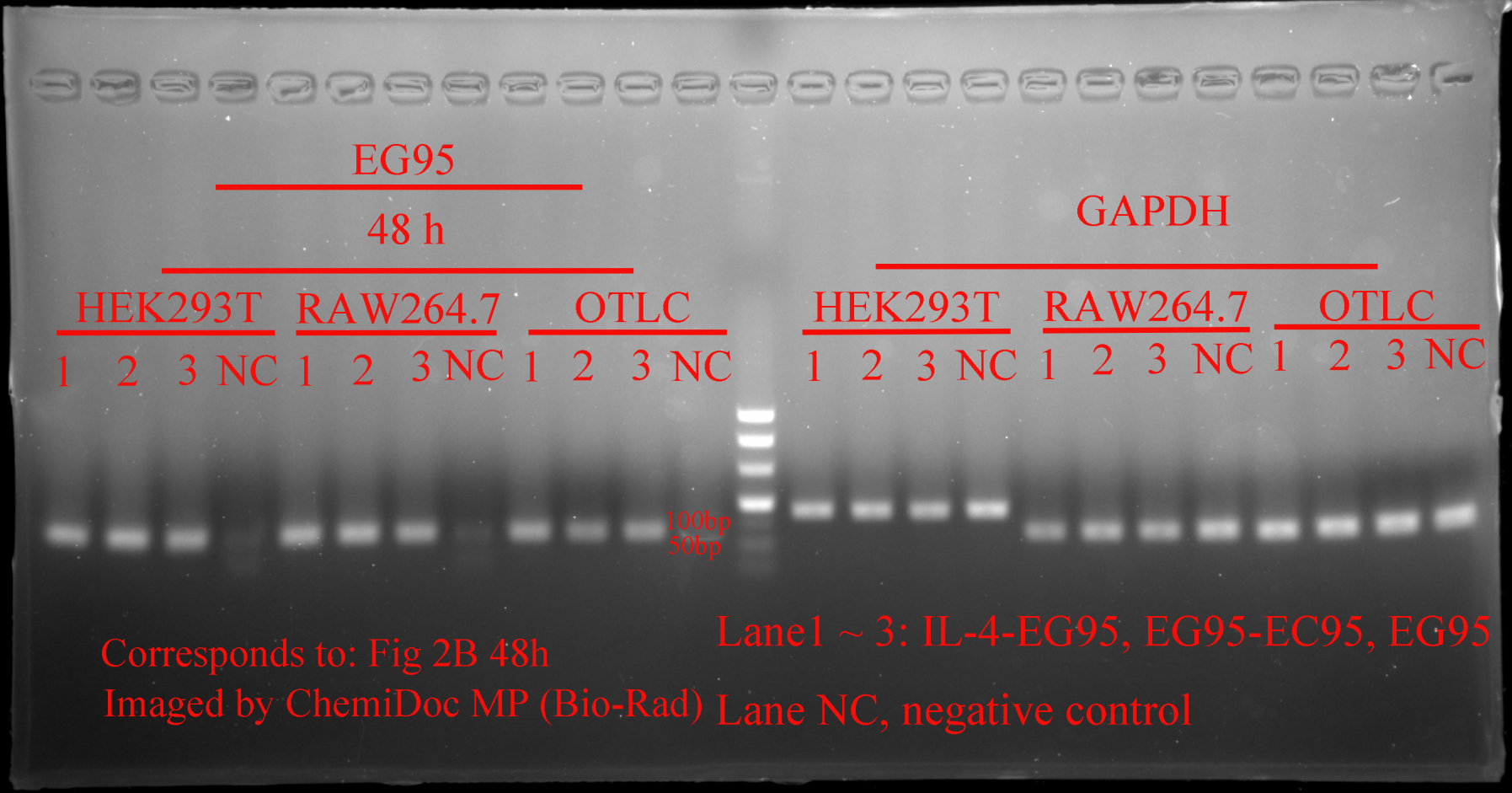
EG95
48 h
HEK293T
RAW264.7
OTLC
1 2 3 NC 1 2 3 NC 1 2 3 NC
100bp
50bp
GAPDH
HEK293T
RAW264.7
OTLC
1 2 3 NC 1 2 3 NC 1 2 3 NC
Corresponds to: Fig 2B 48h
Imaged by ChemiDoc MP (Bio-Rad)
Lane1 ~ 3: IL-4-EG95, EG95-EC95, EG95
Lane NC, negative control

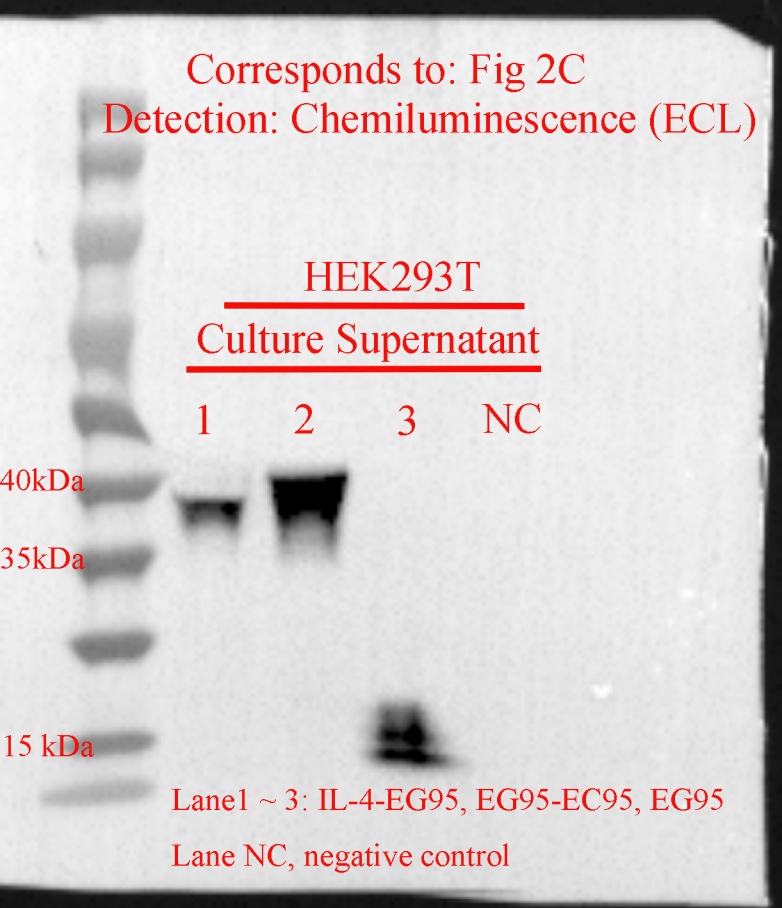
Corresponds to: Fig 2C
Detection: Chemiluminescence (ECL)
HEK293T
Culture Supernatant
1
2
3
NC
40kDa
35kDa
15 kDa
Lane1 ~ 3: IL-4-EG95, EG95-EC95, EG95
Lane NC, negative control

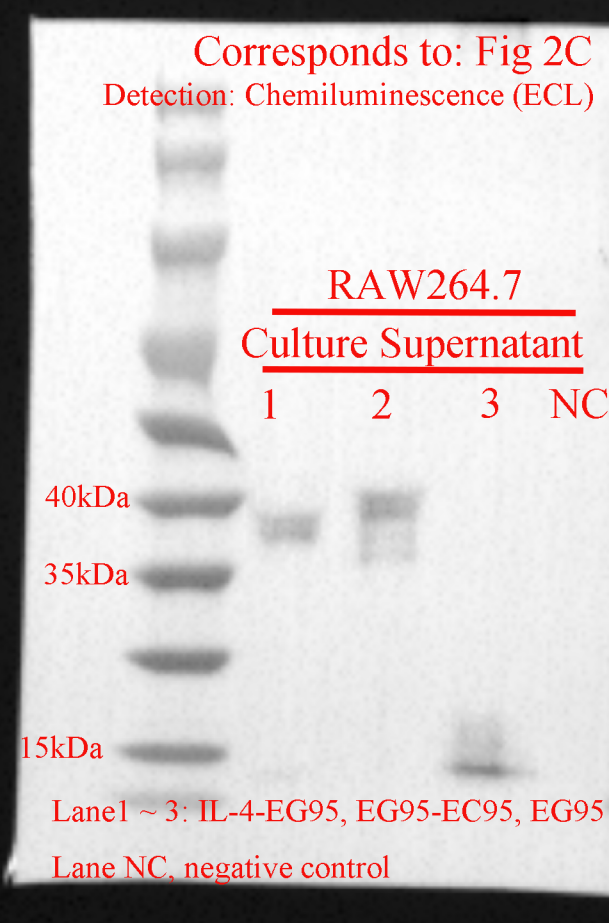

Corresponds to: Fig 2C
Detection: Chemiluminescence (ECL)
RAW264.7
Culture Supernatant
1
2
3
NC
40kDa
35kDa
15kDa
Lane1 ~ 3: IL-4-EG95, EG95-EC95, EG95
Lane NC, negative control

Corresponds to: Fig 2C

Detection: Chemiluminescence (ECL)

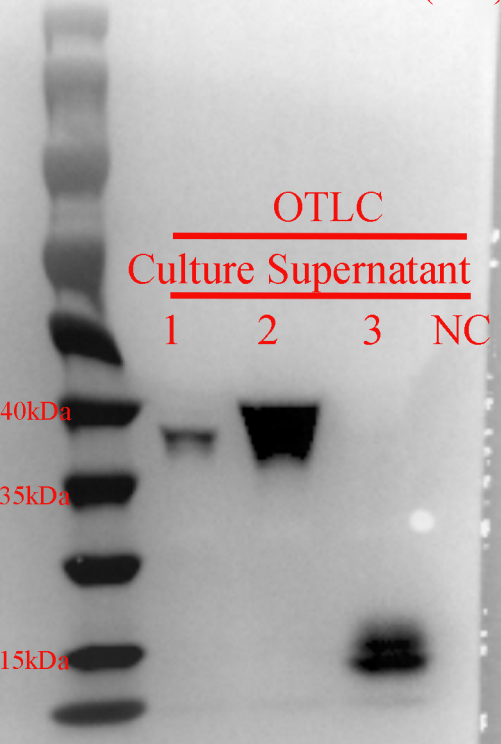

Lane1 ~ 3: IL-4-EG95, EG95-EC95, EG95

Lane NC, negative control

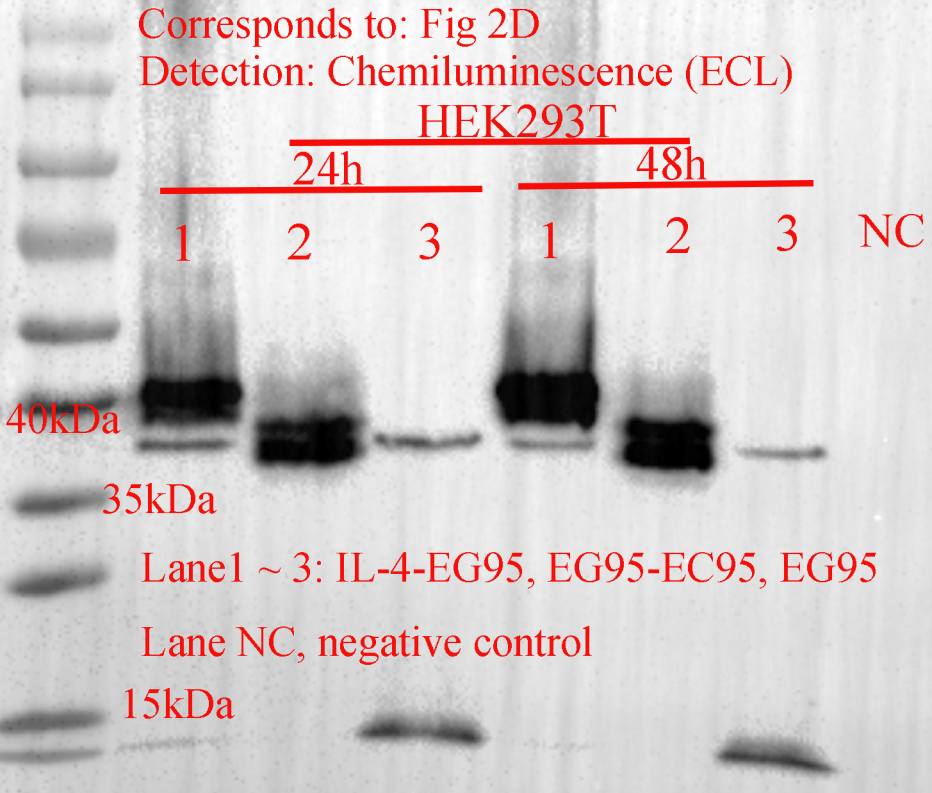
Corresponds to: Fig 2D
Detection: Chemiluminescence (ECL)
HEK293T
24h
48h
1
2
3
1
2
3
NC
40kDa
35kDa
Lane1 ~ 3: IL-4-EG95, EG95-EC95, EG95
Lane NC, negative control
15kDa

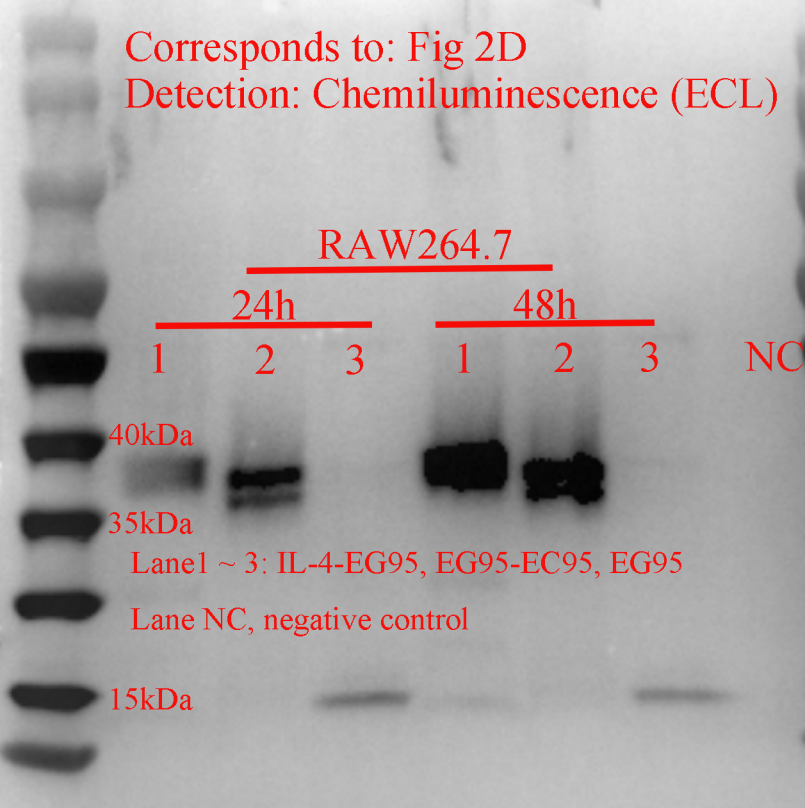
Corresponds to: Fig 2D
Detection: Chemiluminescence (ECL)
RAW264.7
24h
48h
1
2
3
1
2
3
NC
40kDa
35kDa
Lane1 ~ 3: IL-4-EG95, EG95-EC95, EG95
Lane NC, negative control
15kDa

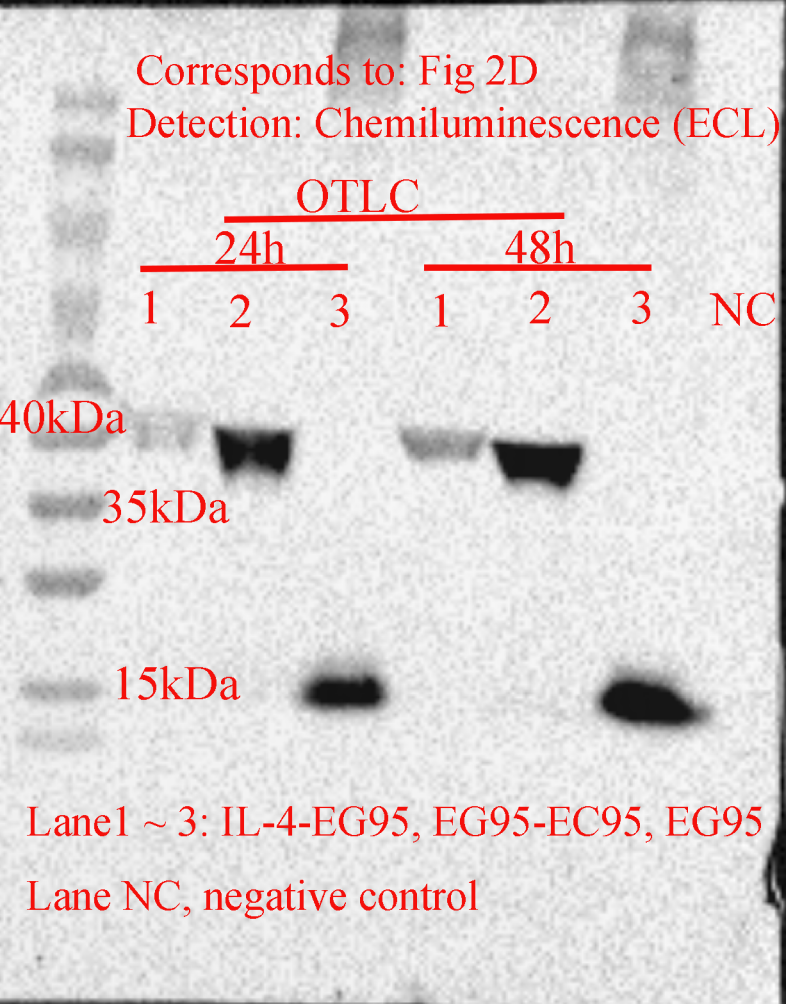
Corresponds to: Fig 2D
Detection: Chemiluminescence (ECL)
OTLC
24h
48h
1
2
3
1
2
3
NC
40kDa
35kDa
15kDa
Lane1 ~ 3: IL-4-EG95, EG95-EC95, EG95
Lane NC, negative control

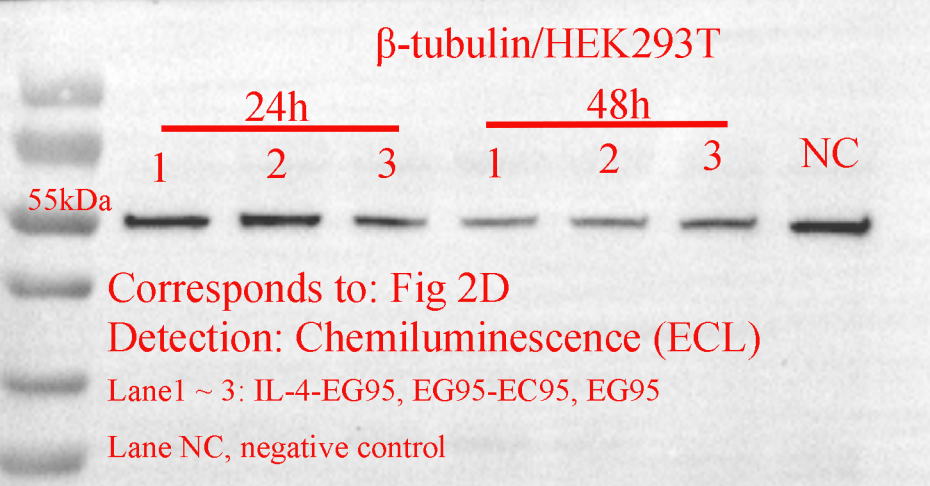
β-tubulin/HEK293T
24h
48h
1
2
3
1
2
3
NC
55kDa
Corresponds to: Fig 2D
Detection: Chemiluminescence (ECL)
Lane1 ~ 3: IL-4-EG95, EG95-EC95, EG95
Lane NC, negative control

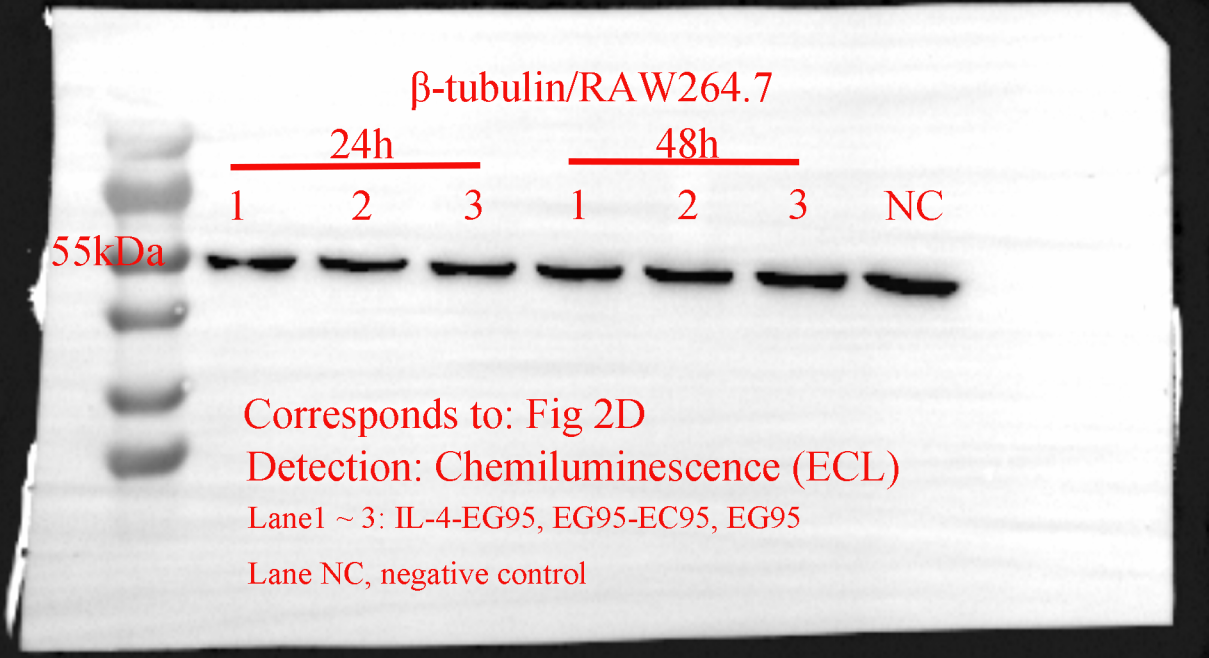
β-tubulin/RAW264.7
24h
48h
1
2
3
1
2
3
NC
55kDa
Corresponds to: Fig 2D
Detection: Chemiluminescence (ECL)
Lane1 ~ 3: IL-4-EG95, EG95-EC95, EG95
Lane NC, negative control

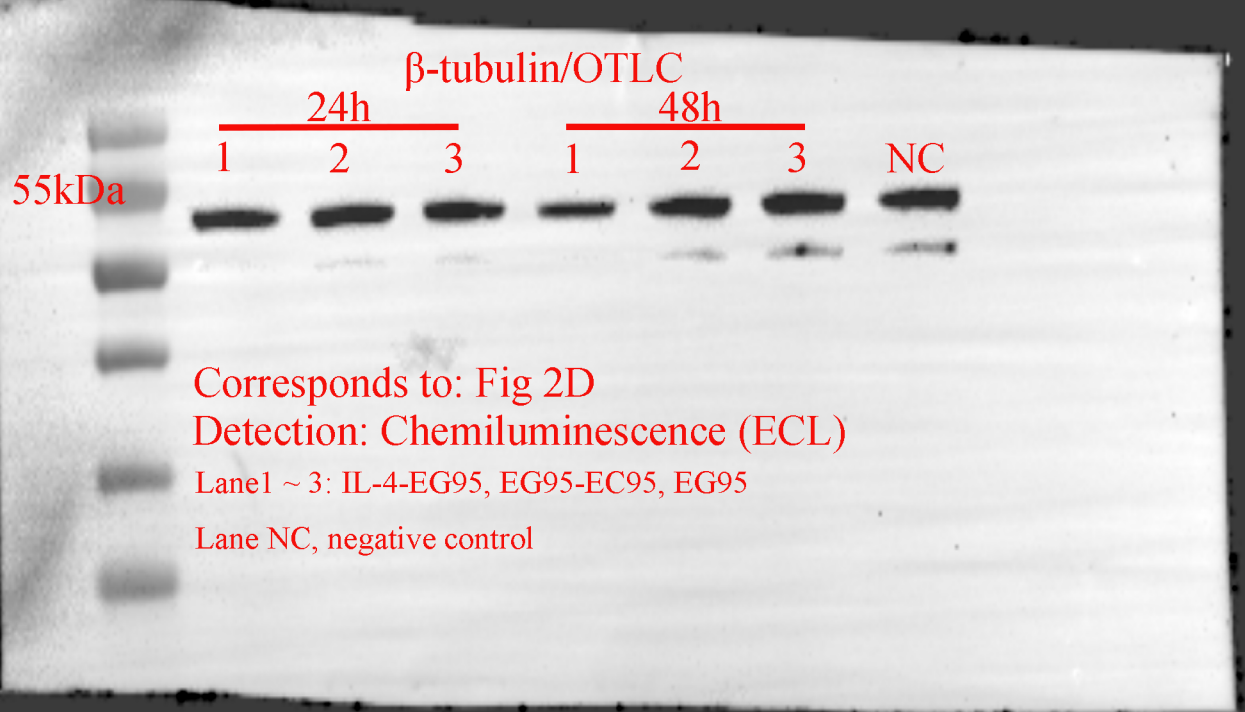
β-tubulin/OTLC
24h
48h
1
2
3
1
2
3
NC
55kDa
Corresponds to: Fig 2D
Detection: Chemiluminescence (ECL)
Lane1 ~ 3: IL-4-EG95, EG95-EC95, EG95
Lane NC, negative control
