# Supplementary material for: Novel EG95-based mRNA vaccines induce long-term protective immunity against echinococcosis
Source: PLoS Pathog. 2026 Oct 9;22(10):e1014659. doi: 10.1371/journal.ppat.1014659 (PMC13674861; doi:10.1371/journal.ppat.1014659)
Supplement: Supplementary file 2 [file ppat.1014659.s002.docx]

| Sample | Z-average (nm) | PDI | Encapsulation efficiency (%) | Zeta potential (mV) | Zeta deviation (mV) |
| --- | --- | --- | --- | --- | --- |
| IL-4-EG95-LNP  EG95-EC95-LNP  EG95-LNP  LNP (empty vector) | 115.8 ± 1.241  114.3 ± 1.573  110.2 ± 0.736  83.5 ± 1.332 | 0.08 ± 0.052  0.10 ± 0.007  0.12 ± 0.003  0.15 ± 0.021 | 96%  98%  95%  —— | -0.997 ± 19.76  -0.345 ± 32.25  -1.461 ± 76.65  -0.677 ± 18.99 | 5.363 ± 6.71  4.893 ± 5.26  5.018 ± 18.99  4.572 ± 5.66 |
